# Von Neumann Stability Analysis of DG-Like and P*N*P*M*-Like Schemes for PDEs with Globally Curl-Preserving Evolution of Vector Fields

**DOI:** 10.1007/s42967-021-00166-x

**Published:** 2022-01-19

**Authors:** Dinshaw S. Balsara, Roger Käppeli

**Affiliations:** 1 Physics and ACMS Departments, University of Notre Dame, Notre Dame, IN USA; 2 Seminar for Applied Mathematics (SAM), Department of Mathematics, ETH Zürich, CH-8092 Zurich, Switzerland

**Keywords:** PDEs, Numerical schemes, Mimetic, Discontinuous Galerkin, 35XX, 35FXX, 35LXX, 35QXX

## Abstract

This paper examines a class of involution-constrained PDEs where some part of the PDE system evolves a vector field whose curl remains zero or grows in proportion to specified source terms. Such PDEs are referred to as curl-free or curl-preserving, respectively. They arise very frequently in equations for hyperelasticity and compressible multiphase flow, in certain formulations of general relativity and in the numerical solution of Schrödinger’s equation. Experience has shown that if nothing special is done to account for the curl-preserving vector field, it can blow up in a finite amount of simulation time. In this paper, we catalogue a class of DG-like schemes for such PDEs. To retain the globally curl-free or curl-preserving constraints, the components of the vector field, as well as their higher moments, must be collocated at the edges of the mesh. They are updated using potentials collocated at the vertices of the mesh. The resulting schemes: (i) do not blow up even after very long integration times, (ii) do not need any special cleaning treatment, (iii) can operate with large explicit timesteps, (iv) do not require the solution of an elliptic system and (v) can be extended to higher orders using DG-like methods. The methods rely on a special curl-preserving reconstruction and they also rely on multidimensional upwinding. The Galerkin projection, highly crucial to the design of a DG method, is now conducted at the edges of the mesh and yields a weak form update that uses potentials obtained at the vertices of the mesh with the help of a multidimensional Riemann solver. A von Neumann stability analysis of the curl-preserving methods is conducted and the limiting CFL numbers of this entire family of methods are catalogued in this work. The stability analysis confirms that with the increasing order of accuracy, our novel curl-free methods have superlative phase accuracy while substantially reducing dissipation. We also show that P*N*P*M*-like methods, which only evolve the lower moments while reconstructing the higher moments, retain much of the excellent wave propagation characteristics of the DG-like methods while offering a much larger CFL number and lower computational complexity. The quadratic energy preservation of these methods is also shown to be excellent, especially at higher orders. The methods are also shown to be curl-preserving over long integration times.

## Introduction

Novel PDEs, important to science and engineering problems are routinely discovered. Many of those PDE systems have involution constraints whose deeper study leads to mimetic numerical solution strategies that retain greater fidelity with the physics of those systems. While involution constraints can come in many forms, the last couple of years have seen the emergence of novel classes of PDEs that place constraints on the evolution of the curl of a vector field. The structure of the evolutionary equation for the vector field of interest is such that it either keeps the curl of the vector field zero, or evolves it in proportion to certain source terms. The former PDEs are referred to as curl-free whereas the latter class of PDEs are referred to as curl-preserving. Many of the hyperbolic systems resulting from the Godunov-Peshkov-Romenski (GPR) formulation for hyperelasticity and compressible multiphase flow with and without surface tension have such curl-preserving update equations (Godunov and Romenski [32], Romenski [40], Romenski et al. [41], Peshkov and Romenski [37, 38], Dumbser et al. [27, 30, 31], Schmidmayer et al. [42]). The equations of general relativity, when cast in the FO-CCZ4 formulation, also have such a structure (Alic et al. [1, 2], Brown et al. [18], Dumbser et al. [29], Dumbser et al. [28]). Similarly, it has recently become possible to recast Schrödinger’s equation in first-order hyperbolic form, and the time-evolution of this very important equation also has curl-preserving constraints (Dhaouadi et al. [25], Busto et al. [19]).

A motivating PDE system would help the reader a lot here. Let us take a simple example involving a fluid with thermal conduction in the GPR formulation. Let us denote the density by $\rho$, the fluid velocity by ${\mathbf{v}}$, the fluid pressure by $P$, the fluid temperature by $T$, the internal thermal energy density by $e$, the total energy density by $E \equiv e + {{\rho {\mathbf{v}}^{2} } \mathord{\left/ {\vphantom {{\rho {\mathbf{v}}^{2} } 2}} \right. \kern-\nulldelimiterspace} 2}$, the thermal impulse by a vector ${\mathbf{J}}$, the heat flux by a vector ${\mathbf{q}}$, and the thermal stress by the second rank tensor ${{\varvec{\upsigma}}}$. The equations for a fluid with thermal conduction can be written as

1a $$ \frac{\partial \rho }{{\partial t}} + \nabla \cdot \left( {\rho {\mathbf{v}}} \right) = 0, $$

1b $$ \frac{{\partial \left( {\rho {\mathbf{v}}} \right)}}{\partial t} + \nabla \cdot \left( {\rho {\mathbf{v}} \otimes {\mathbf{v}} + P{\mathbf{I}} + {{\varvec{\upsigma}}}} \right) = 0, $$

1c $$ \frac{\partial E}{{\partial t}} + \nabla \cdot \left( {\left( {E + P} \right){\mathbf{v}} + {\mathbf{v}} \cdot {{\varvec{\upsigma}}} + {\mathbf{q}}} \right) = 0, $$

1d $$ \frac{{\partial {\mathbf{J}}}}{\partial t} + \nabla \left( {{\mathbf{J}} \cdot {\mathbf{v}} + T} \right) - {\mathbf{v}} \times \left( {\nabla \times {\mathbf{J}}} \right) = - \frac{\rho T}{\tau }{\mathbf{J}}. $$

The identity matrix is denoted by ${\mathbf{I}}$ in the above equations. To complete our description of the above system, we also mention the constitutive relation for the thermal stress tensor $\sigma_{ij} = \rho c_{h}^{2} J_{i} J_{j}$ and another constitutive relation for the thermal conduction vector $q_{i} = \rho Tc_{h}^{2} J_{i}$. Here $c_{h}$ denotes the hyperbolic speed of heat waves, i.e., the second sound. The first three of the four equations in Eq. (1) above are the equations for mass, momentum, and energy conservation for a fluid, with additional contributions from the thermal conduction vector, ${\mathbf{q}}$, and the thermal stress tensor, ${{\varvec{\upsigma}}}$. The fourth equation in Eq. (1) is a novel contribution from the GPR formulation, see (Romenski [40]).

Now focusing on Eq. (1d), let us consider the limit where the relaxation time is very large, so that the source term is irrelevant. Since the vector field ${\mathbf{J}}$ starts off curl-free, it is easy to see that it remains so by considering the remaining two parts of that equation. The first part of the update equation, given by $\nabla \left( {{\mathbf{J}} \cdot {\mathbf{v}} + T} \right)$, is just the gradient of a scalar. Since the curl of a gradient is zero, the first term will not contribute to the curl if none is present initially. The second part of the update equation, given by ${\mathbf{v}} \times \left( {\nabla \times {\mathbf{J}}} \right)$, will also be zero if the vector ${\mathbf{J}}$ is initially curl-free. We see, therefore, that the vector field ${\mathbf{J}}$ stays curl-free if it is initially curl-free in the limit of substantially large value of the relaxation time. Of course, when the relaxation time cannot be ignored, the curl of the vector field does indeed evolve in response to the presence of the stiff source term $- \rho T \, {{\mathbf{J}} \mathord{\left/ {\vphantom {{\mathbf{J}} \tau }} \right. \kern-\nulldelimiterspace} \tau }$. It is important to realize that if the fourth equation in Eq. (1) does not have a consistent discretization, then the curl of the vector field ${\mathbf{J}}$ will only be specified by the accuracy of the numerical method. As a result, even for regions of the flow that should have no thermal conduction, there will at least be a small amount of thermal conduction. This affects the fidelity of the method and its results. For this particular PDE system, Balsara et al. [12] showed via direct comparison that a curl-preserving formulation produces desirable results whereas a direct zone-centered collocation of variables does not. The curl-preserving formulation did not require the inclusion of any additional equations and was able to operate with a robust CFL.

To consider another system, Dumbser et al. [28] analyzed the FO-CCZ4 formulation of general relativity and showed that it has a large number of equations that look like Eq. (1d). The full PDE system is too large to detail here. They showed that zone-centered discretizations simply blow up and found that the only way to mitigate the build-up of circulation in a zone-centered context consisted of adding one extra generalized Lagrange multiplier (GLM) system that evolved an additional vector field and another GLM system that evolved an additional scalar field for *each and every* equation that looks like Eq. (1d). This more than doubles the computational cost along with an imperfect mitigation. Furthermore, the mitigation requires the GLM fields to propagate at speeds that are two or three times larger than the other signal speeds in the problem. As a result, the timestep is reduced by a factor of two or three. Through the description of the above two PDE systems, we see the importance of a consistent, curl-preserving discretization and evolution strategy.

A detailed description of a curl-preserving, finite volume scheme is also provided in Section III.5 of Balsara et al. [12]. The implementation consists of a zone-centered reconstruction of the fluid variables in Eqs. (1a), (1b), (1c) and a curl-preserving reconstruction of the vector field in Eq. (1d). This gives us higher-order spatial accuracy, followed by the application of one-dimensional and two-dimensional Riemann problems at the faces and vertices of the mesh giving us upwinding. Well-known strong stability-preserving Runge-Kutta time stepping then provides higher-order temporal accuracy.

We see, therefore, that many useful PDE systemshave a curl-preserving involution. The simplest example of such a PDE can be written as

2 $$ \frac{{\partial {\mathbf{J}}}}{\partial t} + \nabla \left( {{\mathbf{J}} \cdot {\mathbf{v}} + \varphi \left( \rho \right)} \right) - {\mathbf{v}} \times \left( {\nabla \times {\mathbf{J}}} \right) = {\mathbf{S}}\left( {{\mathbf{J}},\rho } \right). $$

Here ${\mathbf{J}}$ is the vector field of interest and ${\mathbf{v}}$ is some specified velocity field. By “$\rho$” we generically denote other variables that might be part of a larger set of equations to which Eq. (2) belongs. As a result, the potential $\phi \equiv {\mathbf{J}} \cdot {\mathbf{v}} + \varphi \left( \rho \right)$ can also depend on other variables and the source term ${\mathbf{S}}\left( {{\mathbf{J}},\rho } \right)$ depends on “${\mathbf{J}}$” as well as “$\rho$”. By setting the source term “${\mathbf{S}}$” to zero and taking the curl of the above equation, it is easy to see that if we initially have $\left( {\nabla \times {\mathbf{J}}} \right) = 0$, then the structure of the above equation retains $\left( {\nabla \times {\mathbf{J}}} \right) = 0$ for all time; in other words, the evolution of the vector field ${\mathbf{J}}$ is curl-free. If the above equation has a non-zero source term, the evolution of the vector field ${\mathbf{J}}$ would be curl-preserving. In principle, any curl-preserving scheme should be able to reach the curl-free limit as the importance of the source term goes to zero. For the purpose of this study, it is adequate to study curl-free evolution of vector fields because the inclusion or exclusion of the source term does not change the discussion that follows. In other words, the inclusion of source terms requires a separate study of how stiff terms are included in the time-evolution of any hyperbolic PDE, and that is not the point of focus for this paper. Equation (2) shows up quite frequently as a part of many of the hyperbolic systems mentioned before. When Eq. (2) appears in larger systems, it is usually the source of many numerical difficulties.

Equation (2) and the larger systems that include it cannot be evolved with a traditional higher-order Godunov methodology, even when we have accounted for non-conservative products. Indeed, Dumbser et al. [28] have shown that if a classical higher-order Godunov scheme is applied to Eq. (2), the solution blows up rapidly. The blow-up manifests itself as an explosive increase in the curl of the vector field when the simulation is run over long periods of time; please see Fig. 5 of Dumbser et al. [28] which shows the explosive blow-up of the curl of the vector field. The blow-up occurs even when the source terms are zero, indicating that the source terms are not the cause of the difficulty. The problem is not specific to the PDE system considered in that paper because as shown in Boscheri et al. [17], a simple model system based on Eq. (2) will also blow up if treated with a classical higher-order Godunov scheme. A GLM-based cleaning procedure has been developed in Dumbser et al. [28] for suppressing the fictitious numerical build up of the circulation, but it requires greatly increasing the signal speed that is used in the cleaning equations and also adds many more vector fields than necessary. Indeed, Dumbser et al. [28] in their study of a general relativistic system had to use signal speeds in their GLM-style approach that were substantially larger than the speed of light. It is unusual to introduce superluminal signal speeds in a theory that is based on recognizing the speed of light as the maximal signal speed. As a result, GLM cleaning-based schemes force a very strong reduction in timestep, inconsistent with our notions of how the timestep should evolve in a higher-order Godunov scheme. Boscheri et al. [17] obtained curl constraint-preservation but only at the expense of solving an elliptic PDE system at every timestep. Furthermore, the method was restricted to second order of accuracy. This too is inconsistent with our notion that a higher-order Godunov scheme should be time-explicit, easy to solve, and extensible to all higher orders.

In Balsara et al. [12] we first realized that the constrained evolution mandated by Eq. (2) requires a special treatment. Two innovations were introduced in that paper. First, a curl-constraint preserving reconstruction was proposed which requires us to start with a vector field whose components are collocated at the edges of the mesh, and are indeed aligned with the edge directions. Second, it was realized that a multidimensional Riemann solver that is invoked at the vertices of the mesh can indeed give us stabilization through multidimensional upwinding. Coupled with strong stability preserving Runge-Kutta (SSP-RK) timestepping (Shu and Osher [44, 45], Shu [43], Spiteri and Ruuth [46, 47], Gottlieb et al. [33]), we obtained a robust finite volume-based numerical scheme. As a result, both ingredients in the design of a higher-order Godunov scheme—i.e., the reconstruction as well as the Riemann solver—had to be fundamentally rethought when dealing with Eq. (2). These two ingredients proved highly beneficial because Balsara et al. [12] were able to obtain stable finite volume-like schemes for systems that used Eq. (2) which: (i) did not blow up even after very long integration times, (ii) did not need any GLM-style cleaning with its deleterious side-effect of needing very high signal speeds, (iii) could operate with large explicit timesteps, (iv) did not require the solution of an elliptic system and (v) could be extended to higher orders using WENO-like methods. The WENO-like methods draw on ideas from weighted essentially non-oscillatory schemes (Jiang and Shu [35], Balsara and Shu [15], Balsara et al. [9]). As a result, Balsara et al. [12] were able to integrate non-linear hybridization into their novel curl-preserving reconstruction, thus making the curl-preserving reconstruction suitable for use with higher-order Godunov schemes.

In Balsara et al. [12] curl-preserving WENO-like methods were developed for evolving systems that used Eq. (2). We called such methods WENO-like because the reconstruction used many insights from WENO schemes, while being substantially different from traditional, finite volume-based WENO schemes. Recall that a conservation law has a flux form ensuring that the integrated conserved variables in any zone (or connected set of zones) evolve in response to the fluxes at the boundary of that volume. This telescoping property for the fluxes gives the discrete version of the conservation law a globally conservative property. In an entirely analogous fashion, the curl-free schemes presented in Balsara et al. [12] are such that the discrete circulation evaluated over the edges of any face (or collection of faces) depends only on the potentials at the vertices of that facial area. In that sense, the schemes developed in Balsara et al. [12] are globally curl-preserving because they have a telescoping property on the potentials. In that paper, we also presented a von Neumann stability analysis of WENO-like globally curl-preserving schemes, showing that with the increasing order of accuracy the schemes became progressively less dissipative and their dispersion error was also reduced. But it is well-known (Reed and Hill [39], Cockburn and Shu [22–24], Cockburn et al. [20, 21], Liu et al. [36], Zhang and Shu [48]) that finite volume DG schemes have superior wave propagation properties relative to finite volume WENO schemes of comparable order. In their study of DG-like schemes for magnetohydrodynamics and Maxwell’s equations that have one or more constrained, divergence-preserving vector fields, Balsara and Käppeli [10, 11] found a similar trend, and that trend was numerically confirmed by Hazra et al. [34] and Balsara et al. [13]. We may, therefore, expect that curl-preserving DG-like schemes for Eq. (2) should have wave propagation characteristics that are substantially better than curl-preserving WENO-like schemes for Eq. (2) at comparable orders of accuracy. This paper, therefore, has the following three goals.

i) The *first goal* of this paper is to lay out the conceptual foundations for DG-like schemes that preserve the global curl constraint. Recall that a classical DG scheme starts with zone-centered mean values for the flow and endows it with higher moments. These higher moments are then evolved in time by a classical DG scheme. The time evolution of the higher moments is performed consistently with the governing equations. In an exactly analogous fashion, the globally curl-free DG-like schemes start with edge-centered components. These components are then endowed with higher moments whose time-evolution is carried out consistently with the governing equations.
ii) The *second goal* of this paper is to conduct a von Neumann stability analysis of the newly-obtained curl-free DG-like schemes. We use this stability analysis to find the maximum CFL number that is available at all orders up to the fourth order. We also use the stability analysis to show that our new class of DG-like schemes have superior wave propagation characteristics. The curl of a vector field only manifests itself in two or three dimensions. As a result, our von Neumann stability analysis is also two-dimensional. Because the stability analysis is multidimensional by necessity, it is not technically feasible to extend it to very high orders. The computer algebra systems that we use for this stability analysis cannot be pushed beyond fourth order.
iii) Dumbser et al. [26] proposed P*N*P*M* schemes where all the modes that are up to *N*th degree were evolved while all the higher modes up to *M*th degree are reconstructed. The P*N*P*M* schemes had the great advantage that they displayed wave propagation properties almost as good as classical DG schemes of comparable order while permitting substantially larger timesteps. Balsara and Käppeli [10, 11] found a similar trend in their study of divergence-preserving P*N*P*M*-like schemes. Therefore, the *third goal* of this paper is to analyze P*N*P*M*-like schemes that are globally curl-preserving. We intend to show that such P*N*P*M*-like schemes are competitive with their DG-like counterparts at comparable order of accuracy. We also intend to show that the maximum CFL number of P*N*P*M*-like schemes is much larger than that of DG-like schemes.

In this paper we analyze Eq. (2) with $\phi \left( \rho \right) = 0$ and ${\mathbf{S}}\left( {{\mathbf{J}},\rho } \right) = 0$. A constant velocity “${\mathbf{v}}$” is specified. The plan of the paper is as follows. Section 2 introduces a DG-like formulation for a curl-preserving model equation. Section 3 provides the essential ideas behind curl-free and curl-preserving reconstruction while pointing the reader to further literature. Section 4 presents the von Neumann stability analysis. Section 5 presents results from the von Neumann analysis of globally curl-free DG-like schemes. Section 6 presents some numerical results to show that the proposed schemes meet their order of accuracy. Section 7 presents conclusions.

## DG-Like Formulation for the Curl-Preserving Model Equation

Let us consider Eq. (2) to understand its physics, thereby leading us to a curl-preserving DG-like formulation for that equation. Let us first consider Eq. (2) in its curl-free form (i.e., with $\nabla \times {\mathbf{J}} = 0$) since the curl has to be kept mathematically zero in that limit. Since we are considering a minimalist system, we can also take $\varphi \left( \rho \right) = 0$ because we are ignoring any further PDEs in the system. We write the vector components in two-dimensions as ${\mathbf{J}} = \left( {J^{x} ,J^{y} } \right)$ and assume a constant velocity ${\mathbf{v}} = \left( {{\text{v}}_{x} ,{\text{v}}_{y} } \right)$, so that the dot product becomes ${\mathbf{J}} \cdot {\mathbf{v}} = {\text{v}}^{x} J^{x} + {\text{v}}^{y} J^{y}$. The equations that we have to solve can be written as

3 $$ \frac{{\partial J^{x} }}{\partial t} + \frac{{\partial \left( {{\text{v}}^{x} J^{x} + {\text{v}}^{y} J^{y} } \right)}}{\partial x} = 0{;}\quad \frac{{\partial J^{y} }}{\partial t} + \frac{{\partial \left( {{\text{v}}^{x} J^{x} + {\text{v}}^{y} J^{y} } \right)}}{\partial y} = 0\quad {\text{with the constraint }} {\frac{{\partial J^{y} }}{\partial x} - \frac{{\partial J^{x} }}{\partial y}} = 0. $$

Using the constraint in each of the two equations, they can be written as

4 $$ \frac{{\partial J^{x} }}{\partial t} + {\text{v}}^{x} \frac{{\partial J^{x} }}{\partial x} + {\text{v}}^{y} \frac{{\partial J^{x} }}{\partial y} = 0{;}\quad \frac{{\partial J^{y} }}{\partial t} + {\text{v}}^{x} \frac{{\partial J^{y} }}{\partial x} + {\text{v}}^{y} \frac{{\partial J^{y} }}{\partial y} = 0. $$

**Fig. 1:**
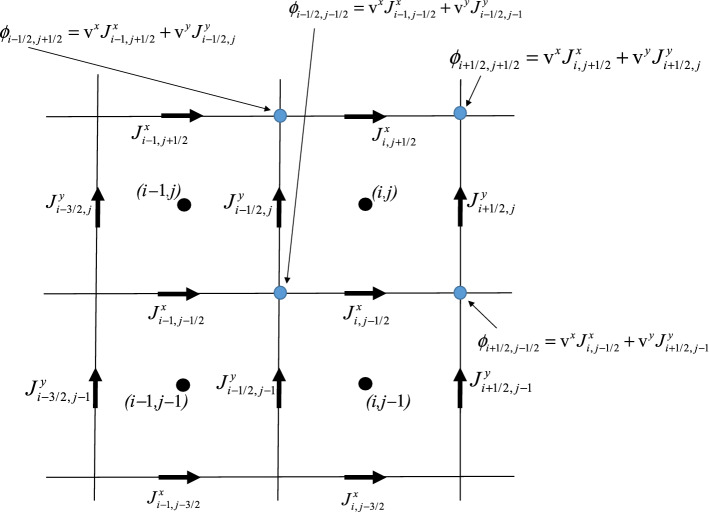
The components of the curl-free vector field around the four zones centered around (*i*, *j*), (*i* − 1, *j*), (*i* − 1, *j* − 1), and (*i*, *j* − 1). A first order curl-free reconstruction is used in this figure; though the use of a higher order reconstruction of the vector field is also possible. The multidimensionally upwinded potentials at the vertices of the zone (*i*, *j*) are also shown for the situation where both components of the velocity are positive. Again, while the potentials are shown explicitly for the first order case in the figure, a higher order reconstruction will yield more accurate potentials at the vertices of the mesh

In other words, Eq. (4) tells us that we should be able to advect two components of a vector field. However, importantly, we should be able to carry out this advection of the vector field in a fashion that preserves the curl-free aspect of the vector field over each zone. We can only carry out such an advection if the component $J^{x}$ is collocated at the *x*-edges of the mesh and the component $J^{y}$ is collocated at the *y*-edges of the mesh. Furthermore, the potential, ${\text{v}}^{x} J^{x} + {\text{v}}^{y} J^{y}$, should be collocated at the vertices of the mesh, as shown in Fig. 1. Furthermore, Eq. (4) shows us that the potential should be obtained via multidimensional upwinding at the vertices of the mesh. For this simple example, multidimensional upwinding can be enforced visually depending on the direction of the velocity field. For example, Fig. 1 shows the multidimensionally upwinded potentials, when both components of the velocity are positive. On a mesh with zone sizes $\Delta x$ and $\Delta y$ in the *x*- and *y*-directions, the curl-free update equations at the first order (with positive velocity components) can be written as

5 $$ \left\{ \begin{gathered} \frac{{\partial J_{i,j + 1/2}^{x} }}{\partial t} +  {\frac{{\left( {{\text{v}}^{x} J_{i,j + 1/2}^{x} + {\text{v}}^{y} J_{i + 1/2,j}^{y} } \right) - \left( {{\text{v}}^{x} J_{i - 1,j + 1/2}^{x} + {\text{v}}^{y} J_{i - 1/2,j}^{y} } \right)}}{\Delta x}} { = 0;} \hfill \\ \frac{{\partial J_{i + 1/2,j}^{y} }}{\partial t} +  {\frac{{\left( {{\text{v}}^{x} J_{i,j + 1/2}^{x} + {\text{v}}^{y} J_{i + 1/2,j}^{y} } \right) - \left( {{\text{v}}^{x} J_{i,j - 1/2}^{x} + {\text{v}}^{y} J_{i + 1/2,j - 1}^{y} } \right)}}{\Delta y}}  = 0. \hfill \\ \end{gathered} \right.$$

Equation (5) shows us that the collocation described in Fig. 1 is crucial for maintaining a curl-free update and it will have to be built into our curl-free DG-like scheme. The use of identical potentials at each vertex of the mesh allows us to claim that the discrete version of the curl-free constraint

6 $$ \frac{{J_{i + 1/2,j}^{y} - J_{i - 1/2,j}^{y} }}{\Delta x} - \frac{{J_{i,j + 1/2}^{x} - J_{i,j - 1/2}^{x} }}{\Delta y} = 0 $$

is preserved forever for each and every zone of the mesh. In other words, the update equations are globally curl-free. Equation (5) also shows us the importance of multidimensional upwinding providing a unique potential at each vertex of the mesh. To prove that Eq. (6) follows from Eq. (5) at all orders of accuracy, please write out equations like Eq. (5) for $J_{i + 1/2,j}^{y}$, $J_{i - 1/2,j}^{y}$, $J_{i,j + 1/2}^{x}$ and $J_{i,j - 1/2}^{x}$ in Fig. 1. Please write the equations out in terms of the potentials $\phi_{i + 1/2,j + 1/2}$, $\phi_{i - 1/2,j + 1/2}$, $\phi_{i + 1/2,j - 1/2}$, and $\phi_{i - 1/2,j - 1/2}$ shown at the vertices in Fig. 1. Notice that these potentials can be made as accurate as we desire by the use of a higher-order reconstruction of the vector field. The pairwise cancellation will show that Eq. (6) is satisfied at all orders—in other words, we have a mimetic scheme. More generally, when Eq. (2) is incorporated into a larger PDE system, the multidimensional Riemann solver (Balsara [3–6], Balsara et al. [8], Balsara and Dumbser [7], Balsara et al. [16], Balsara and Nkonga [14]) provides us with multidimensional upwinding consistent with the waves propagating in all directions.

The process of designing a higher-order DG-like scheme consists of endowing the primal variables with higher-order moments and then evolving those moments consistently with the governing equations. Consider the zone (*i*,* j*) in Fig. 1. We center the coordinates at the zone center so that the two-dimensional zone has extent $\left[ { - {{\Delta x} \mathord{\left/ {\vphantom {{\Delta x} 2}} \right. \kern-\nulldelimiterspace} 2},{{\Delta x} \mathord{\left/ {\vphantom {{\Delta x} 2}} \right. \kern-\nulldelimiterspace} 2}} \right] \times \left[ { - {{\Delta y} \mathord{\left/ {\vphantom {{\Delta y} 2}} \right. \kern-\nulldelimiterspace} 2},{{\Delta y} \mathord{\left/ {\vphantom {{\Delta y} 2}} \right. \kern-\nulldelimiterspace} 2}} \right]$. At the right *y*-edge and the top *x*-edge of the zone we assert the moments up to fourth order as

7 $$ \left\{ \begin{gathered} J^{y} \left( {y,t} \right) = J_{0}^{y} \left( t \right) + J_{y}^{y} \left( t \right)\left( {\frac{y}{\Delta y}} \right) + J_{yy}^{y} \left( t \right)\left( {\left( {\frac{y}{\Delta y}} \right)^{2} - \frac{1}{12}} \right) + J_{yyy}^{y} \left( t \right)\left( {\left( {\frac{y}{\Delta y}} \right)^{3} - \frac{3}{20}\left( {\frac{y}{\Delta y}} \right)} \right){;} \hfill \\ J^{x} \left( {x,t} \right) = J_{0}^{x} \left( t \right) + J_{x}^{x} \left( t \right)\left( {\frac{x}{\Delta x}} \right) + J_{xx}^{x} \left( t \right)\left( {\left( {\frac{x}{\Delta x}} \right)^{2} - \frac{1}{12}} \right) + J_{xxx}^{x} \left( t \right)\left( {\left( {\frac{x}{\Delta x}} \right)^{3} - \frac{3}{20}\left( {\frac{x}{\Delta x}} \right)} \right). \hfill \\ \end{gathered} \right. $$

In a DG-like scheme, the modes in Eq. (7) become time-evolutionary. If only the linear part of Eq. (7) is retained, we get a second-order DG-like scheme; if the quadratic part of Eq. (7) is retained, we get a third-order DG-like scheme; if all the terms of Eq. (7) are retained, we get a fourth-order DG-like scheme. Equation (7) makes it easy to see the trial functions that are asserted in each of the mesh edges. We will use test functions identical to the trial functions. Let us first write the Galerkin projection in the abstract and then specialize it to Eq. (7). It is useful to realize that a traditional finite volume DG method is derived from a Gauss’ law-based vector identity

$$ \nabla \cdot \left( {\psi \, {\mathbf{F}}} \right) = \psi \, \nabla \cdot {\mathbf{F}} + {\mathbf{F}} \cdot \nabla \psi . $$

From the one-dimensional gradients involved in Eq. (3) we realize that the DG-like methods that we seek depend on the product rule for derivatives applied one-dimensionally. In other words, we rely on the identity

$$ \frac{{\partial \left( {\psi \phi } \right)}}{\partial x} = \psi \frac{\partial \phi }{{\partial x}} + \phi \frac{\partial \psi }{{\partial x}}. $$

In the above equation, “$\psi$” is a test function at the mesh edges. Applying the above identity to the *y*-component of Eq. (2) we can make the Galerkin projection in the *y*-edge of the mesh as follows:

8 $$ \begin{aligned} & \frac{\partial }{\partial t}\left( {\int\nolimits_{y = - \Delta y/2}^{\Delta y/2} {\psi \left( y \right)J^{y} \left( {y,t} \right){\text{d}}y} } \right) + \psi \left( {y = \Delta y/2} \right)\phi \left( {y = \Delta y/2} \right) - \psi \left( {y = - \Delta y/2} \right)\phi \left( {y = - \Delta y/2} \right) \\ & \quad -  {\int\nolimits_{y = - \Delta y/2}^{\Delta y/2} {\psi^{'} \left( y \right)\phi \left( y \right){\text{d}}y} }  +  {\int\nolimits_{y = - \Delta y/2}^{\Delta y/2} {\psi \left( y \right){\text{v}}_{x} \left( {\nabla \times {\mathbf{J}}} \right)_{z} {\text{d}}y} }  =  {\int\nolimits_{y = - \Delta y/2}^{\Delta y/2} {\psi \left( y \right)S_{y} \left( {{\mathbf{J}},\rho } \right){\text{d}}y} } . \\ \end{aligned} $$

Here $\psi \left( y \right)$ is a test function at the *y*-edge of Fig. 1. Similarly, applying the above identity to the *x*-component of Eq. (2) we get

9 $$ \begin{aligned} & \frac{\partial }{\partial t}\left( {\int\nolimits_{x = - \Delta x/2}^{\Delta x/2} {\psi \left( x \right)J^{x} \left( {x,t} \right){\text{d}}x} } \right) + \psi \left( {x = \Delta x/2} \right)\phi \left( {x = \Delta x/2} \right) - \psi \left( {x = - \Delta x/2} \right)\phi \left( {x = - \Delta x/2} \right) \\ & \quad -  {\int\nolimits_{x = - \Delta x/2}^{\Delta x/2} {\psi^{'} \left( x \right)\phi \left( x \right){\text{d}}x} }  -  {\int\nolimits_{x = - \Delta x/2}^{\Delta x/2} {\psi \left( x \right){\text{v}}_{y} \left( {\nabla \times {\mathbf{J}}} \right)_{z} {\text{d}}x} } =  {\int\nolimits_{x = - \Delta x/2}^{\Delta x/2} {\psi \left( x \right)S_{x} \left( {{\mathbf{J}},\rho } \right){\text{d}}x} } . \\ \end{aligned} $$

There is not much going on in Eqs. (8) and (9) other than an integration by parts along with a one-dimensional Galerkin projection. However, in the next paragraph we interpret the above two equations to bring out the physics of the situation.

Equation (8) allows us to write the update equations for the evolutionary modes of $J^{y} \left( {y,t} \right)$ as

10a $$ \frac{{{\text{d}}J_{0}^{y} \left( t \right)}}{{{\text{d}}t}} + \frac{1}{\Delta y}\left[ {\phi^{**} \left( {y = \Delta y/2} \right) - \phi^{**} \left( {y = - \Delta y/2} \right)} \right] + \left\langle {{\text{v}}_{x} \left( {\nabla \times {\mathbf{J}}} \right)_{z} } \right\rangle = \left\langle {S_{y} \left( {{\mathbf{J}}^{*} ,\rho^{*} } \right)} \right\rangle, $$

10b $$ \begin{aligned} & \frac{1}{12}\frac{{{\text{d}}J_{y}^{y} \left( t \right)}}{{{\text{d}}t}} + \frac{1}{2\Delta y}\left[ {\phi^{**} \left( {y = \Delta y/2} \right) + \phi^{**} \left( {y = - \Delta y/2} \right)} \right] - \frac{1}{\Delta y}\left\langle {\phi^{*} \left( y \right)} \right\rangle \\ & \quad + \left\langle {\left( {\frac{y}{\Delta y}} \right){\text{v}}_{x} \left( {\nabla \times {\mathbf{J}}} \right)_{z} } \right\rangle = \left\langle {\left( {\frac{y}{\Delta y}} \right)S_{y} \left( {{\mathbf{J}}^{*} ,\rho^{*} } \right)} \right\rangle ,\\ \end{aligned} $$

10c $$ \begin{aligned} & \frac{1}{180}\frac{{{\text{d}}J_{yy}^{y} \left( t \right)}}{{{\text{d}}t}} + \frac{1}{6\Delta y}\left[ {\phi^{**} \left( {y = \Delta y/2} \right) - \phi^{**} \left( {y = - \Delta y/2} \right)} \right] - \frac{2}{\Delta y}\left\langle {\left( {\frac{y}{\Delta y}} \right)\phi^{*} \left( y \right)} \right\rangle \\ & \quad + \left\langle {\left( {\left( {\frac{y}{\Delta y}} \right)^{2} - \frac{1}{12}} \right){\text{v}}_{x} \left( {\nabla \times {\mathbf{J}}} \right)_{z} } \right\rangle = \left\langle {\left( {\left( {\frac{y}{\Delta y}} \right)^{2} - \frac{1}{12}} \right)S_{y} \left( {{\mathbf{J}}^{*} ,\rho^{*} } \right)} \right\rangle, \\ \end{aligned} $$

10d $$ \begin{aligned} & \frac{1}{2\,800}\frac{{{\text{d}}J_{yyy}^{y} \left( t \right)}}{{{\text{d}}t}} + \frac{1}{20\Delta y}\left[ {\phi^{**} \left( {y = \Delta y/2} \right) + \phi^{**} \left( {y = - \Delta y/2} \right)} \right] - \frac{3}{\Delta y}\left\langle {\left( {\left( {\frac{y}{\Delta y}} \right)^{2} - \frac{1}{20}} \right)\phi^{*} \left( y \right)} \right\rangle \\ & \quad + \left\langle {\left( {\left( {\frac{y}{\Delta y}} \right)^{3} - \frac{3}{20}\left( {\frac{y}{\Delta y}} \right)} \right){\text{v}}_{x} \left( {\nabla \times {\mathbf{J}}} \right)_{z} } \right\rangle = \left\langle {\left( {\left( {\frac{y}{\Delta y}} \right)^{3} - \frac{3}{20}\left( {\frac{y}{\Delta y}} \right)} \right)S_{y} \left( {{\mathbf{J}}^{*} ,\rho^{*} } \right)} \right\rangle . \\ \end{aligned} $$

The angled brackets, 〈〉, represent line integrated averages of sufficiently high order within a *y*-edge (and later, similarly, for the *x*-edge). The potentials $$\phi^{**}$$ with the double star superscripts denote the potentials obtained at the mesh vertices using a multidimensional Riemann solver. The potentials $$\phi^{*} \left( y \right)$$ with the single star superscripts denote potentials obtained by the application of one-dimensional Riemann solvers at the *y*-edge of the desired zone. These one-dimensional Riemann solvers may be invoked at multiple quadrature points at the *y*-edge so that the terms $$\left\langle {\phi^{*} \left( y \right)} \right\rangle$$ and $$\left\langle {\left( {\frac{y}{\Delta y}} \right)\phi^{*} \left( y \right)} \right\rangle$$ are accurately evaluated. The source terms have a similar interpretation so that the $${\mathbf{J}}^{*}$$ and $$\rho^{*}$$ variables in $$S_{y} \left( {{\mathbf{J}}^{*} ,\rho^{*} } \right)$$ are obtained from one-dimensional Riemann solvers. We see from Eq. (10a) that the update of the mean value, $$J_{0}^{y} \left( t \right)$$, will be curl-preserving and approach curl-free evolution in the limit where the source term tends to zero. Equations (10b)–(10d) show the same type of body terms that arise in a classical DG scheme due to the integration by parts with a test function with the key difference that they are now applied to the edges of the mesh. The terms involving $$\left( {\nabla \times {\mathbf{J}}} \right)_{z}$$ in Eq. (10) also have a special interpretation. These terms are exactly zero when the evolution is curl-free. When the evolution is only curl-preserving, these terms will be proportional to the discrete circulation around the zone, but only if a curl-preserving reconstruction from Balsara et al. [16] is used. From each of the two sides of a two-dimensional mesh, we can obtain terms that provide $$\left( {\nabla \times {\mathbf{J}}} \right)_{z}$$. The resulting $$\left\langle {{\text{v}}_{x} \left( {\nabla \times {\mathbf{J}}} \right)_{z} } \right\rangle$$ in Eq. (10a) is therefore an arithmetic average of the curl evaluated from either side of that edge. We make analogous interpretations for the terms with $$\left( {\nabla \times {\mathbf{J}}} \right)_{z}$$ in Eqs. (10b)–(10d).

Equation (9) allows us to write the update equations for the evolutionary modes of $J^{x} \left( {x,t} \right)$ as

11a $$ \frac{{{\text{d}}J_{0}^{x} \left( t \right)}}{{{\text{d}}t}} + \frac{1}{\Delta x}\left[ {\phi^{**} \left( {x = \Delta x/2} \right) - \phi^{**} \left( {x = - \Delta x/2} \right)} \right] - \left\langle {{\text{v}}_{y} \left( {\nabla \times {\mathbf{J}}} \right)_{z} } \right\rangle = \left\langle {S_{x} \left( {{\mathbf{J}}^{*} ,\rho^{*} } \right)} \right\rangle, $$

11b $$ \begin{aligned} & \frac{1}{12}\frac{{{\text{d}}J_{x}^{x} \left( t \right)}}{{{\text{d}}t}} + \frac{1}{2\Delta x}\left[ {\phi^{**} \left( {x = \Delta x/2} \right) + \phi^{**} \left( {x = - \Delta x/2} \right)} \right] - \frac{1}{\Delta x}\left\langle {\phi^{*} \left( x \right)} \right\rangle \\ & \quad - \left\langle {\left( {\frac{x}{\Delta x}} \right){\text{v}}_{y} \left( {\nabla \times {\mathbf{J}}} \right)_{z} } \right\rangle = \left\langle {\left( {\frac{x}{\Delta x}} \right)S_{x} \left( {{\mathbf{J}}^{*} ,\rho^{*} } \right)} \right\rangle, \\ \end{aligned} $$

11c $$ \begin{aligned} & \frac{1}{180}\frac{{{\text{d}}J_{xx}^{x} \left( t \right)}}{{{\text{d}}t}} + \frac{1}{6\Delta x}\left[ {\phi^{**} \left( {x = \Delta x/2} \right) - \phi^{**} \left( {x = - \Delta x/2} \right)} \right] - \frac{2}{\Delta x}\left\langle {\left( {\frac{x}{\Delta x}} \right)\phi^{*} \left( x \right)} \right\rangle \\ & \quad - \left\langle {\left( {\left( {\frac{x}{\Delta x}} \right)^{2} - \frac{1}{12}} \right){\text{v}}_{y} \left( {\nabla \times {\mathbf{J}}} \right)_{z} } \right\rangle = \left\langle {\left( {\left( {\frac{x}{\Delta x}} \right)^{2} - \frac{1}{12}} \right)S_{x} \left( {{\mathbf{J}}^{*} ,\rho^{*} } \right)} \right\rangle,  \\ \end{aligned} $$

11d $$ \begin{aligned} & \frac{1}{2\,800}\frac{{{\text{d}}J_{xxx}^{x} \left( t \right)}}{{{\text{d}}t}} + \frac{1}{20\Delta x}\left[ {\phi^{**} \left( {x = \Delta x/2} \right) + \phi^{**} \left( {x = - \Delta x/2} \right)} \right] - \frac{3}{\Delta x}\left\langle {\left( {\left( {\frac{x}{\Delta x}} \right)^{2} - \frac{1}{20}} \right)\phi^{*} \left( x \right)} \right\rangle \\ & \quad - \left\langle {\left( {\left( {\frac{x}{\Delta x}} \right)^{3} - \frac{3}{20}\left( {\frac{x}{\Delta x}} \right)} \right){\text{v}}_{y} \left( {\nabla \times {\mathbf{J}}} \right)_{z} } \right\rangle = \left\langle {\left( {\left( {\frac{x}{\Delta x}} \right)^{3} - \frac{3}{20}\left( {\frac{x}{\Delta x}} \right)} \right)S_{x} \left( {{\mathbf{J}}^{*} ,\rho^{*} } \right)} \right\rangle . \\ \end{aligned} $$

The interpretation of the terms in Eq. (11) mirrors that of Eq. (10). In Eq. (11), the angled brackets, 〈〉, represent line integrated averages of sufficiently high order within the *x*-edge.

In this work, we are interested in analyzing curl-free evolution, with the result that all terms with $\left( {\nabla \times {\mathbf{J}}} \right)_{z}$, $S_{x} \left( {{\mathbf{J}}^{*} ,\rho^{*} } \right)$ and $S_{y} \left( {{\mathbf{J}}^{*} ,\rho^{*} } \right)$ can be set to zero in Eqs. (10) and (11). Without the support of a larger PDE system, it is not possible to specify the source terms. Nevertheless, it is important for the reader to understand what a curl-preserving reconstruction is. We illustrate the same for the simplest of cases in the next section.

## Curl-Preserving Reconstruction

**Fig. 2:**
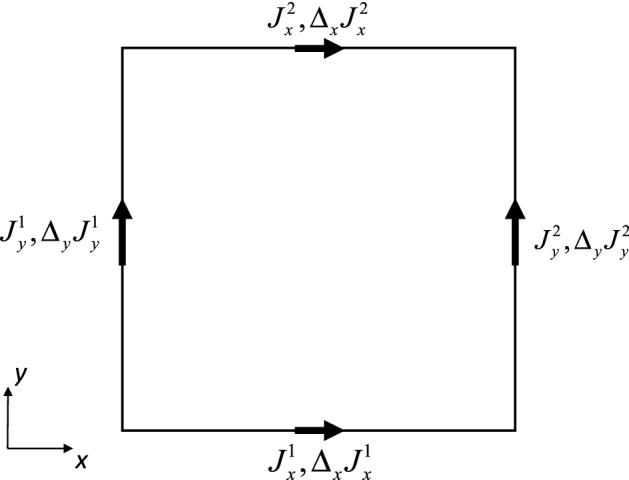
Collocation of vector components along the edges of a two-dimensional control volume. As evaluated over the edges of the square element, the discrete circulation is fully specified. The mean value of the vector components and their linear variation are shown along each edge, in keeping with a second order accurate reconstruction scheme. The reconstruction problem for a curl-preserving reconstruction consists of obtaining a polynomial-based vector field that matches the specified mean circulation in the zone while simultaneously matching the edge values within each zone

Equations (10) and (11) show that we need a reconstruction strategy within a zone that matches the vector components and their higher moments in the edges of the mesh. This is needed because we want the scheme to be globally curl-preserving. Consequently, each edge, as seen by its abutting zones, will have the same component of the vector field as well as its higher moments. Equation (2), as well as Eqs. (10) and (11) show that when the discrete circulation evaluated around a zone is small, it should make proportionately small contributions to the edges via the $\left( {\nabla \times {\mathbf{J}}} \right)_{z}$-dependent terms. In other words, the curl of the reconstructed vector field should match the discrete circulation (evaluated around each zone) as well as its higher moments. Figure 2 shows us an example of how this works in two dimensions and at second order. In Balsara et al. [12] we have presented two and three-dimensional versions of such a reconstruction strategy at several orders. Here we just show some details for the second-order case so that the reader may appreciate the core ideas as they are presented in one self-contained place.

Consider the vector field that is shown in Fig. 2. We consider the zone to be a unit square spanning $\left[ { - {1 \mathord{\left/ {\vphantom {1 2}} \right. \kern-\nulldelimiterspace} 2},{1 \mathord{\left/ {\vphantom {1 2}} \right. \kern-\nulldelimiterspace} 2}} \right] \times \left[ { - {1 \mathord{\left/ {\vphantom {1 2}} \right. \kern-\nulldelimiterspace} 2},{1 \mathord{\left/ {\vphantom {1 2}} \right. \kern-\nulldelimiterspace} 2}} \right]$. The modes at the left and right *y*-edges are given by

12 $$ J_{y}^{1} + \left( {\Delta_{y} J_{y}^{1} } \right)y\quad {\text{and}}\quad J_{y}^{2} + \left( {\Delta_{y} J_{y}^{2} } \right)y. $$

The modes in the bottom and top *x*-edges are given by

13 $$ J_{x}^{1} + \left( {\Delta_{x} J_{x}^{1} } \right)x\quad {\text{and}}\quad J_{x}^{2} + \left( {\Delta_{x} J_{x}^{2} } \right)x. $$

We can write the equations of a vector field which matches the values of the components and their linear variation within each edge as follows:

14 $$ \left\{ \begin{aligned} J^{x} \left( {x,y} \right) & = \left[ {J_{x}^{1} + \left( {\Delta_{x} J_{x}^{1} } \right)x} \right]\left( {\frac{1}{2} - y} \right) + \left[ {J_{x}^{2} + \left( {\Delta_{x} J_{x}^{2} } \right)x} \right]\left( {\frac{1}{2} + y} \right) + a_{yy} \left( {1 - 4y^{2} } \right){;} \\ J^{y} \left( {x,y} \right) & = \left[ {J_{y}^{1} + \left( {\Delta_{y} J_{y}^{1} } \right)y} \right]\left( {\frac{1}{2} - x} \right) + \left[ {J_{y}^{2} + \left( {\Delta_{y} J_{y}^{2} } \right)y} \right]\left( {\frac{1}{2} + x} \right) + b_{xx} \left( {1 - 4x^{2} } \right). \\ \end{aligned} \right. $$

Note that the coefficients $a_{yy}$ and $b_{xx}$ are needed for curl constraint satisfaction. The discrete circulation within the zone of interest is given by $\left[ {J_{x}^{1} - J_{x}^{2} + J_{y}^{2} - J_{y}^{1} } \right]$, with the result that we can obtain the higher modes of that variable up to linear variation and write it as

15 $$ R^{z} \left( {x,y} \right) = \left[ {J_{x}^{1} - J_{x}^{2} + J_{y}^{2} - J_{y}^{1} } \right] + \left( {\Delta_{x} R^{z} } \right)x + \left( {\Delta_{y} R^{z} } \right)y. $$

Notice that if the discrete circulation $\left[ {J_{x}^{1} - J_{x}^{2} + J_{y}^{2} - J_{y}^{1} } \right]$ is zero, its variation will also be zero, so that we get $\left( {\Delta_{x} R^{z} } \right)$ and $\left( {\Delta_{y} R^{z} } \right)$ as zero values. If the discrete circulation $\left[ {J_{x}^{1} - J_{x}^{2} + J_{y}^{2} - J_{y}^{1} } \right]$ is small, its variation as represented by $\left( {\Delta_{x} R^{z} } \right)$ and $\left( {\Delta_{y} R^{z} } \right)$ will also be proportionately small. We want to fix the coefficients $a_{yy}$ and $b_{xx}$ so that the curl of the vector field in Eq. (14) exactly matches Eq. (15). This is obtained by setting

16 $$ b_{xx} = \frac{1}{8}\left[ { - \left( {\Delta_{x} R^{z} } \right) + \left( {\Delta_{x} J_{x}^{1} } \right) - \left( {\Delta_{x} J_{x}^{2} } \right)} \right]{;}\quad a_{yy} = \frac{1}{8}\left[ {\left( {\Delta_{y} R^{z} } \right) + \left( {\Delta_{y} J_{y}^{1} } \right) - \left( {\Delta_{y} J_{y}^{2} } \right)} \right]. $$

This shows that the reconstructed vector field in Eq. (14) has been reconstructed in curl-preserving fashion. Therefore, the growth of the curl in Eqs. (10) and (11) is perfectly well-controlled and consistent with the PDE in Eq. (2). Furthermore, when the discrete circulation is exactly zero, the $\left( {\nabla \times {\mathbf{J}}} \right)_{z}$-dependent terms in Eqs. (10) and (11) contribute absolutely nothing. In other words, the curl-free limit is exactly retrieved by our choice of reconstruction and discretization.

We can use Eq. (14) along with Eq. (16) to write the reconstructed curl-preserving vector field in terms of an orthonormal set of modes that span the zone itself. Projecting Eq. (14) into an orthonormal basis made of tensor product Legendre polynomials, we get

17 $$ \begin{aligned} J^{x} \left( {x,y} \right) & = \left[ {{{\left( {J_{x}^{1} + J_{x}^{2} } \right)} \mathord{\left/ {\vphantom {{\left( {J_{x}^{1} + J_{x}^{2} } \right)} {2 + {{\left( {\left( {\Delta_{y} R^{z} } \right) + \left( {\Delta_{y} J_{y}^{1} } \right) - \left( {\Delta_{y} J_{y}^{2} } \right)} \right)} \mathord{\left/ {\vphantom {{\left( {\left( {\Delta_{y} R^{z} } \right) + \left( {\Delta_{y} J_{y}^{1} } \right) - \left( {\Delta_{y} J_{y}^{2} } \right)} \right)} {12}}} \right. \kern-\nulldelimiterspace} {12}}}}} \right. \kern-\nulldelimiterspace} {2 + {{\left( {\left( {\Delta_{y} R^{z} } \right) + \left( {\Delta_{y} J_{y}^{1} } \right) - \left( {\Delta_{y} J_{y}^{2} } \right)} \right)} \mathord{\left/ {\vphantom {{\left( {\left( {\Delta_{y} R^{z} } \right) + \left( {\Delta_{y} J_{y}^{1} } \right) - \left( {\Delta_{y} J_{y}^{2} } \right)} \right)} {12}}} \right. \kern-\nulldelimiterspace} {12}}}}} \right] + \left[ {{{\left( {\left( {\Delta_{x} J_{x}^{1} } \right) + \left( {\Delta_{x} J_{x}^{2} } \right)} \right)} \mathord{\left/ {\vphantom {{\left( {\left( {\Delta_{x} J_{x}^{1} } \right) + \left( {\Delta_{x} J_{x}^{2} } \right)} \right)} 2}} \right. \kern-\nulldelimiterspace} 2}} \right]x + \left[ { - J_{x}^{1} + J_{x}^{2} } \right]y \\ & \quad + \left[ { - {{\left( {\left( {\Delta_{y} J_{y}^{1} } \right) - \left( {\Delta_{y} J_{y}^{2} } \right) + \left( {\Delta_{y} R^{z} } \right)} \right)} \mathord{\left/ {\vphantom {{\left( {\left( {\Delta_{y} J_{y}^{1} } \right) - \left( {\Delta_{y} J_{y}^{2} } \right) + \left( {\Delta_{y} R^{z} } \right)} \right)} 2}} \right. \kern-\nulldelimiterspace} 2}} \right]\left( {y^{2} - {1 \mathord{\left/ {\vphantom {1 {12}}} \right. \kern-\nulldelimiterspace} {12}}} \right) + \left[ { - \left( {\Delta_{x} J_{x}^{1} } \right) + \left( {\Delta_{x} J_{x}^{2} } \right)} \right]xy, \\ J^{y} \left( {x,y} \right) & = \left[ {{{\left( {J_{y}^{1} + J_{y}^{2} } \right)} \mathord{\left/ {\vphantom {{\left( {J_{y}^{1} + J_{y}^{2} } \right)} 2}} \right. \kern-\nulldelimiterspace} 2} + {{\left( { - \left( {\Delta_{x} R^{z} } \right) + \left( {\Delta_{x} J_{x}^{1} } \right) - \left( {\Delta_{x} J_{x}^{2} } \right)} \right)} \mathord{\left/ {\vphantom {{\left( { - \left( {\Delta_{x} R^{z} } \right) + \left( {\Delta_{x} J_{x}^{1} } \right) - \left( {\Delta_{x} J_{x}^{2} } \right)} \right)} {12}}} \right. \kern-\nulldelimiterspace} {12}}} \right] + \left[ { - J_{y}^{1} + J_{y}^{2} } \right]x + \left[ {{{\left( {\left( {\Delta_{y} J_{y}^{1} } \right) + \left( {\Delta_{y} J_{y}^{2} } \right)} \right)} \mathord{\left/ {\vphantom {{\left( {\left( {\Delta_{y} J_{y}^{1} } \right) + \left( {\Delta_{y} J_{y}^{2} } \right)} \right)} 2}} \right. \kern-\nulldelimiterspace} 2}} \right]y \\ & \quad + \left[ { - {{\left( {\left( {\Delta_{x} J_{x}^{1} } \right) - \left( {\Delta_{x} J_{x}^{2} } \right) - \left( {\Delta_{x} R^{z} } \right)} \right)} \mathord{\left/ {\vphantom {{\left( {\left( {\Delta_{x} J_{x}^{1} } \right) - \left( {\Delta_{x} J_{x}^{2} } \right) - \left( {\Delta_{x} R^{z} } \right)} \right)} 2}} \right. \kern-\nulldelimiterspace} 2}} \right]\left( {x^{2} - {1 \mathord{\left/ {\vphantom {1 {12}}} \right. \kern-\nulldelimiterspace} {12}}} \right) + \left[ { - \left( {\Delta_{y} J_{y}^{1} } \right) + \left( {\Delta_{y} J_{y}^{2} } \right)} \right]xy. \\ \end{aligned} $$

Notice that Eq. (17) shows us that there is a transcription from the modes that we use in a curl-preserving DG-like scheme to the modes that we would use for a finite volume-based DG scheme. We see that the modes of a curl-preserving DG-like scheme just combine differently, consistent with the constraints, to give us the modes in a traditional, finite volume-based DG scheme. This ensures that the order property is always retained. Note though that curl constraint-preservation results in some higher-order modes in Eq. (17) that would not be present in a classical second-order finite volume-based DG scheme. Therefore, the reverse transcription, i.e., going from the modes of a finite volume-based DG scheme of a certain order to the modes of a curl-preserving DG-like scheme of the same order, does not hold. At second order, one cannot make much from this transcription because all the coefficients in Eq. (17) are fully determined. However, as shown in Section II.4 of Balsara et al. [12], at fourth order and beyond, some of the modes of the higher-order curl-preserving reconstruction have to be obtained volumetrically while others are obtained from the edges of the mesh.

## Von Neumann Stability Analysis of Curl-Free DG Schemes: Second-Order Example

The von Neumann stability analysis of a DG scheme can be performed in two different styles. The first is to convert the DG equations into a finite-difference-like form (Liu et al. [36], Zhang and Shu [48], Balsara and Käppeli [10]). The second approach is to identify the minimal number of modes, endow them with harmonic variation and then to directly carry out the stability analysis on the primal variables of the DG scheme (Balsara and Käppeli [11]). The latter approach works very well because it quickly allows us to identify the smallest number of variables that should be retained in a constraint-preserving DG scheme.

**Fig. 3:**
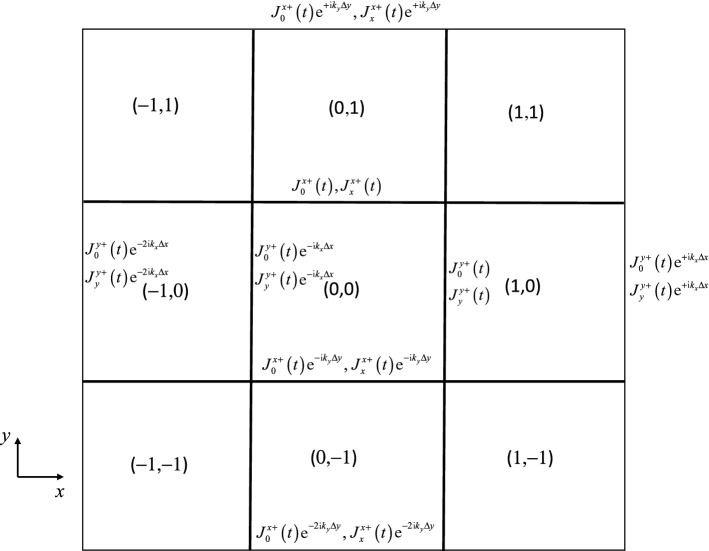
Relation of the facially collocated Fourier modes associated with the curl-free vector field with one another across the different mesh faces. These Fourier modes, and their analogues at all the other faces in the figure, are used for carrying out the von Neumann stability analysis

Here we describe the basic ingredients that go into carrying out a von Neumann stability analysis for a second-order accurate curl-free DG-like scheme. Please focus on Fig. 3. In the right *y*-edge of the central zone we identify the modes $J_{0}^{y + } \left( t \right)$ and $J_{y}^{y + } \left( t \right)$ as the mean *y*-component of the vector field and its linear variation in the *y*-direction. Similarly, in the top *x*-edge of the central zone we identify the modes $J_{0}^{x + } \left( t \right)$ and $J_{x}^{x + } \left( t \right)$ as the mean *x*-component of the vector field and its linear variation in the *x*-direction. In the spirit of a DG scheme, the modes are endowed with time-dependence. In the spirit of a harmonic variation, we assume rectangular zones of sizes $\Delta x$ and $\Delta y$ so that the Fourier modes vary as ${\text{e}}^{{ - {\text i}\left( {k_{x} x + k_{y} y} \right)}}$ where the wave vector is given by $\left( {k_{x} ,k_{y} } \right)$. In fact, we simplify even further by assuming square zones in most parts of this paper. Because we use Fourier modes in a von Neumann stability analysis, the modes along the left *y*-edge are related to the modes along the right *y*-edge. Similarly, the modes along the bottom *x*-edge are related to the modes along the top *x*-edge. The relationship goes as follows

18 $$ \left\{ \begin{aligned} & J_{0}^{y - } \left( t \right) = J_{0}^{y + } \left( t \right){\text{e}}^{{ - {\text i}k_{x} \Delta x}} {;}\quad J_{y}^{y - } \left( t \right) = J_{y}^{y + } \left( t \right){\text{e}}^{{ - {\text i}k_{x} \Delta x}} {;} \\ & J_{0}^{x - } \left( t \right) = J_{0}^{x + } \left( t \right){\text{e}}^{{ - {\text i}k_{y} \Delta y}} {;}\quad J_{x}^{x - } \left( t \right) = J_{x}^{x + } \left( t \right){\text{e}}^{{ - {\text i}k_{y} \Delta y}} . \\ \end{aligned} \right.$$

Figure 3 shows even further inter-relationships among the modes that reside along the edges once the Fourier modal variation is assumed. At first blush it would seem that each zone in Fig. 3 has four independent pieces of information given by $J_{0}^{y + } \left( t \right)$, $J_{y}^{y + } \left( t \right)$, $J_{0}^{x + } \left( t \right)$ and $J_{x}^{x + } \left( t \right)$. However, because of the curl-free constraint, there are only three independent pieces of information. This becomes apparent when we use Eq. (18) to write the discrete curl-free condition in zone $\left( {i,j} \right)$ of Fig. 3 as follows:

19 $$ \frac{{J_{0}^{y + } \left( t \right) - J_{0}^{y + } \left( t \right){\text{e}}^{{ - {\text i}k_{x} \Delta x}} }}{\Delta x} - \frac{{J_{0}^{x + } \left( t \right) - J_{0}^{x + } \left( t \right){\text{e}}^{{ - {\text i}k_{y} \Delta y}} }}{\Delta y} = 0\quad \Leftrightarrow \quad J_{0}^{x + } \left( t \right) = J_{0}^{y + } \left( t \right)\frac{\Delta y}{{\Delta x}}\frac{{1 - {\text{e}}^{{ - {\text i}k_{x} \Delta x}} }}{{1 - {\text{e}}^{{ - {\text i}k_{y} \Delta y}} }}. $$

As a result of Eq. (19), the only independent variables in the von Neumann stability analysis of a second-order accurate, curl-free DG-like scheme are $J_{0}^{y + } \left( t \right)$, $J_{y}^{y + } \left( t \right)$ and $J_{x}^{x + } \left( t \right)$. This simplifies the analysis considerably.

Figure 3 shows how the facial modes at all the mesh faces are inter-related because of the Fourier modes and their spatial variation. As a result, the curl-free reconstruction of the vector field in each zone of Fig. 3 can be symbolically carried out using a computer algebra system. Equations (10a), (10b), and (11b) (in their curl-free forms) can then again be symbolically expressed using the same computer algebra system. As a result, we obtain expressions for the time rate of change of $J_{0}^{y + } \left( t \right)$, $J_{y}^{y + } \left( t \right)$, and $J_{x}^{x + } \left( t \right)$ that can be written in terms of $J_{0}^{y + } \left( t \right)$, $J_{y}^{y + } \left( t \right)$, and $J_{x}^{x + } \left( t \right)$. In other words, we have reduced the problem of evaluating a single stage in the multistage RK-timestepping to the problem of obtaining a linear system of ODEs that look as follows:

20 $$ \frac{\partial }{\partial t}\left( {\begin{array}{*{20}c} {J_{0}^{y + } \left( t \right)} \\ {J_{y}^{y + } \left( t \right)} \\ {J_{x}^{x + } \left( t \right)} \\ \end{array} } \right) = \left( {\begin{array}{*{20}c} {A_{11} } & {A_{12} } & {A_{13} } \\ {A_{21} } & {A_{22} } & {A_{23} } \\ {A_{31} } & {A_{32} } & {A_{33} } \\ \end{array} } \right)\left( {\begin{array}{*{20}c} {J_{0}^{y + } \left( t \right)} \\ {J_{y}^{y + } \left( t \right)} \\ {J_{x}^{x + } \left( t \right)} \\ \end{array} } \right). $$

The nine coefficients in the matrix shown in Eq. (20) depend only on the wave numbers $k_{x}$ and $k_{y}$, the velocities ${\text{v}}_{x}$ and ${\text{v}}_{y}$, and the zone sizes $\Delta x$ and $\Delta y$. They are explicitly given in Appendix A. We can also formally define the vector of unknowns as $${\mathbf{V}}\left( t \right) = \left( {\begin{array}{*{20}c} {J_{0}^{y + } \left( t \right),} & {J_{y}^{y + } \left( t \right),} & {J_{x}^{x + } \left( t \right)} \\ \end{array} } \right)^{{\text{T}}}$$. As a result, Eq. (20) can be formally written as $\partial_{t} {\mathbf{V}}\left( t \right) = {\mathbf{A}} \, {\mathbf{V}}\left( t \right)$, where “**A**” is the 3 × 3 matrix shown in Eq. (20).

We then discretize Eq. (20) in time with an explicit *m*-stage Runge-Kutta scheme having a timestep $\Delta t$ of the form

21 $$ \left\{ \begin{aligned} &{\mathbf{V}}^{\left( 0 \right)} = {\mathbf{V}}\left( {t^{n} } \right), \hfill \\ &{\mathbf{V}}^{\left( i \right)} = \sum\limits_{k = 0}^{i - 1} {\left( {\alpha_{i,k} {\mathbf{I}} + \Delta t\beta_{i,k} {\mathbf{A}}} \right){\mathbf{V}}^{\left( k \right)} } \quad {\text{for }}i = 1, \cdots ,m, \hfill \\ & {\mathbf{V}}\left( {t^{n + 1} } \right) = {\mathbf{V}}^{\left( m \right)} . \hfill \\ \end{aligned} \right. $$

Here “**I**” is the identity matrix. The expressions for the coefficients $\alpha_{i,k}$ and $\beta_{i,k}$ can be found in Gottlieb et al. [33] and also Spiteri and Ruuth [46, 47]. Given the linearity of our DG scheme, we can write the time update as

22 $$ {\mathbf{V}}\left( {t^{n + 1} } \right) = {\mathbf{G}} \, {\mathbf{V}}\left( {t^{n} } \right). $$

Here “**G**” is known as the amplification matrix of the scheme. It depends on the coefficients of the Runge-Kutta scheme, on the timestep $\Delta t$ and the matrix “**A**” from Eq. (20). For the second-order SSP-RK scheme we can write the amplification matrix as

23 $$ {\mathbf{G}} = {\mathbf{I}} + \Delta t{\mathbf{A}} + \frac{{\Delta t^{2} }}{2}{\mathbf{A}}^{2} . $$

Likewise, for the third-order SSP-RK scheme we can write the amplification matrix as

24 $$ {\mathbf{G}} = {\mathbf{I}} + \Delta t{\mathbf{A}} + \frac{{\Delta t^{2} }}{2}{\mathbf{A}}^{2} + \frac{{\Delta t^{3} }}{3!}{\mathbf{A}}^{3} . $$

In the next section, we will use this amplification matrix to devise our von Neumann stability analysis. This completes our description of the mathematics associated with the von Neumann stability analysis in second order. Higher orders can be done similarly.

## Results from the von Neumann Stability Analysis of Globally Curl-Free DG-Like Schemes

By taking a close look at Eq. (3) we realize that the von Neumann stability analysis depends on the angle that the velocity vector $\left( {{\text{v}}_{x} ,{\text{v}}_{y} } \right)$ makes with respect to the *x*-axis of the mesh. Furthermore, Eqs. (18) and (19) show us that the von Neumann stability analysis also depends on the angle that the wave vector $\left( {k_{x} ,k_{y} } \right)$ makes with respect to the velocity vector $\left( {{\text{v}}_{x} ,{\text{v}}_{y} } \right)$. For this reason, the stability analysis depends on multiple parameters. Besides, owing to the fact that the curl only manifests itself in two or more dimensions, it has to be multidimensional. For all of these reasons, we have only been able to carry out a von Neumann stability analysis for curl-free WENO-like, P*N*P*M*-like and DG-like schemes up to fourth order of accuracy. However, we realize that such a stability analysis that is done in two dimensions and for a full scheme can give us a wealth of information, and that information is catalogued in the ensuing two sub-sections.

The first insight that we would like to extract from such a stability analysis is the maximal CFL number for which the scheme is stable. For Eq. (3) the Fourier modes are indeed propagating with the velocity vector; therefore, the velocity vector sets the signal speed. For each choice of the spatial accuracy, we can choose a temporal accuracy for our SSP-RK scheme that is comparable or greater than the spatial accuracy. The upshot is that for each choice of the spatial and temporal accuracy, we can identify a maximal CFL number. Please realize that this involves sweeping through all velocities in two dimensions and for each choice of the velocity we have to sweep over all the wavenumbers that are permitted on the mesh. Stable CFL numbers are identified as the ones for which all possible wave vectors return an amplification matrix all of whose eigenvalues have an absolute value that is less than or equal to unity. Such a study of the maximal CFL number is documented in Sect. 5.1.

DG-like schemes can be very accurate, even when they are compared to their WENO-like counterparts. But we need to visually appreciate that. For that reason, we choose velocity vectors that make angles of 0°, 15°, 30°, and 45° to the mesh. For each of those velocity vectors, we sweep through all possible angles that the wave vector can make with respect to the velocity. This allows us to visualize the dissipation and dispersion errors of the schemes that we analyze. This information is shown in Sect. 5.2.

### Maximal CFL Numbers from Stability Analysis

**Fig. 4:**
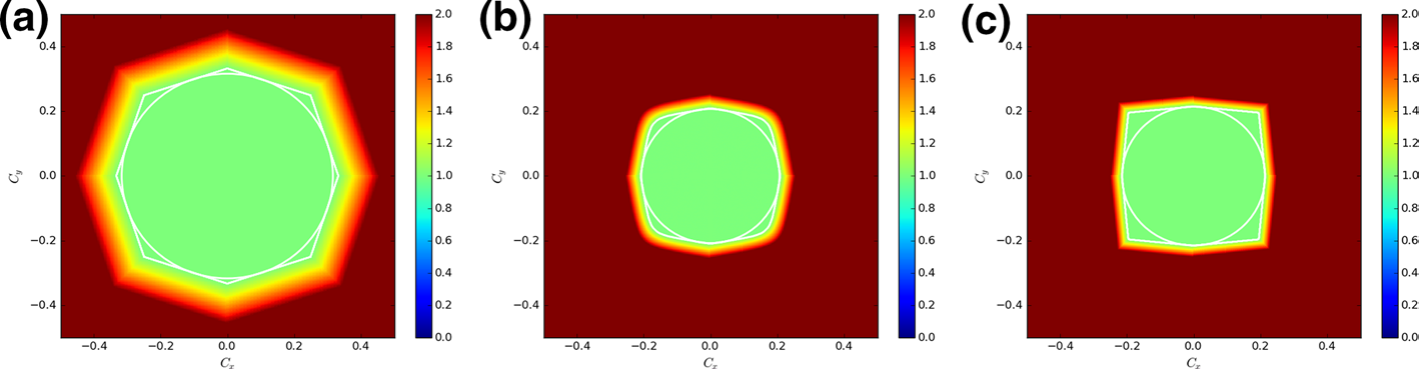
The domain of stability for **a** a second order in space and time curl-free DG-like scheme that uses SSK-RK2 timestepping, **b** a third order in space and time curl-free DG-like scheme that uses SSK-RK3 timestepping, and **c** a fourth order in space and time curl-free DG-like scheme that uses SSP-RK(5,4) timestepping. The CFL numbers in the *x*- and *y*-directions are denoted by *C*_*x*_ and *C*_*y*_, and the color coding shows the absolute value of the largest eigenvalue of the amplification matrix. The white polygon identifies the full domain of stability and the white circle identifies the largest circle that can be fit within the domain of stability. The radius of the white circle, therefore, gives us the maximal CFL number

We identify the CFL number in each of the two directions by $C_{x} = {{{\text{v}}_{x} \Delta t} \mathord{\left/ {\vphantom {{{\text{v}}_{x} \Delta t} {\Delta x}}} \right. \kern-\nulldelimiterspace} {\Delta x}}$ and $C_{y} = {{{\text{v}}_{y} \Delta t} \mathord{\left/ {\vphantom {{{\text{v}}_{y} \Delta t} {\Delta y}}} \right. \kern-\nulldelimiterspace} {\Delta y}}$. For each CFL number in either of the two directions, we sweep over all possible wave numbers $\left( {k_{x} \Delta x,k_{y} \Delta y} \right) \in \left[ { - {\uppi \mathord{\left/ {\vphantom {\uppi 2}} \right. \kern-\nulldelimiterspace} 2},{\uppi \mathord{\left/ {\vphantom {\uppi 2}} \right. \kern-\nulldelimiterspace} 2}} \right] \times \left[ { - {\uppi \mathord{\left/ {\vphantom {\uppi 2}} \right. \kern-\nulldelimiterspace} 2},{\uppi \mathord{\left/ {\vphantom {\uppi 2}} \right. \kern-\nulldelimiterspace} 2}} \right]$. Figure 4 shows a colorized plot of the eigenvector of the amplification matrix with the largest absolute value for second, third, and fourth-order curl-free DG-like schemes. The white polygons in Fig. 4 identify the domain of stability for which the absolute value described above is less than or equal to unity. The white circles in Fig. 4 are the largest circles that can be inscribed in the polygons. The radii of those circles give us the largest effective CFL number that we should use for each of those DG-like schemes. Figure 4 is intended to give us a glimpse of the process to find the largest effective CFL number that we can find from our von Neumann stability analysis.

Figure 4 corresponds to a situation where the order of temporal accuracy of our SSP-RK time stepping schemes indeed matched the spatial accuracy of the DG-like discretization. But we can use several possible SSP-RK schemes with each DG-like discretization, as long as the temporal accuracy is at least as large as the spatial accuracy. Table 1 shows the largest effective CFL number for a range of curl-free DG-like spatial discretizations and a range of temporal accuracies. In all cases, SSP-RK schemes were used for the temporal update. We see that for each scheme, an increasing temporal accuracy results in a larger effective CFL number, a result that conforms to the findings of Zhang and Shu [48] and Liu et al. [36].

**Table 1:** The largest effective CFL number for a range of curl-free DG-like spatial discretizations and a range of temporal accuracies

	*P* = 0	*P* = 1	*P* = 2	*P* = 3
RK1	0.707 1	–	–	–
SSP-RK2	0.707 1	0.316 2	–	–
SSP-RK3	0.888 4	0.390 6	0.206 9	–
SSP-RK(5,4)	1.549 5	0.636 7	0.340 1	0.214 3
In all cases, SSP-RK schemes were used for the temporal update				

Because we have erected the machinery of the von Neumann stability analysis, we can also use it to analyze the largest effective CFL number when other spatial discretizations are used. For example, when we retain only the time-evolution of the zeroth mode in Eqs. (10a) and (11a), we get a family of curl-free WENO-like schemes. For those schemes, all the higher modes, up to the desired order of accuracy, have to be reconstructed. Table 2 shows the largest effective CFL number for a range of curl-free WENO-like spatial discretizations and a range of temporal accuracies. In all cases, SSP-RK schemes were used for the temporal update. As expected, we see that the curl-free WENO-like schemes have CFL numbers much larger than the curl-free DG-like schemes.

**Table 2:** The largest effective CFL number for a range of curl-free WENO-like spatial discretizations and a range of temporal accuracies

	P0P0	P0P1	P0P2	P0P3
RK1	0.707 1	–	–	–
SSP-RK2	0.707 1	0.707 1	–	–
SSP-RK3	0.888 4	0.831 8	1.150 7	–
SSP-RK(5,4)	1.549 5	1.225 2	1.485 9	1.304 0
In all cases, SSP-RK schemes were used for the temporal update				

Table 3 shows the largest effective CFL number for a range of curl-free P1P*M*-like spatial discretizations and a range of temporal accuracies. In addition to retaining the time evolution of the modes in Eqs. (10a) and (11a), such schemes also evolve the first moments from Eqs. (10b) and (11b). As before, SSP-RK schemes were used for the temporal update. Comparing the CFL numbers from Table 3 to those from Tables 1 and 2, we see that curl-free P1P*M*-like schemes give us CFL numbers that are somewhat smaller than those of their WENO counterparts but substantially larger than their DG counterparts. We will further see in the next sub-section that P1P*M*-like schemes retain their first moments and that gives them dissipation and dispersion properties that are closer to their DG counterparts. The physical reason for that is because the linear mode retains most of the variation in the zone; consequently, much of the accuracy is retained. We, therefore, understand why curl-free P1P*M*-like schemes retain an important utilitarian position in the full range of schemes studied here.

**Table 3:** The largest effective CFL number for a range of curl-free P1P*M*-like spatial discretizations and a range of temporal accuracies

	P1P2	P1P3
SSP-RK3	0.390 3	–
SSP-RK(5,4)	0.626 0	0.679 9
In all cases, SSP-RK schemes were used for the temporal update		

This completes our study of the CFL number of curl-free DG-like schemes and their cousins.

### Dissipation and Dispersion Properties of DG and P*N*P*M* Schemes

We now wish to study the dissipation and dispersion properties of the curl-free WENO-like, P*N*P*M*-like, and DG-like schemes. We will study these properties for second, third, and fourth order, so that we have a clear understanding of the improved wave propagation properties of these schemes with the increasing order. By the same token, we will also be able to inter-compare between the WENO-like, P*N*P*M*-like, and DG-like schemes. We expect that retaining more moments and evolving them consistently with the governing PDE, should give us schemes with improved wave propagation characteristics. For all the data shown in this sub-section, the temporal accuracy was made to match the spatial accuracy. While the von Neumann stability analysis for curl-free WENO-like schemes was already documented in Balsara et al. [12], we present it again here so that one can inter-compare with the P*N*P*M*-like and DG-like schemes. The von Neumann stability analysis of the curl-free P*N*P*M*-like and DG-like schemes is being presented for the very first time here.

In each instance, we choose velocity vectors that make angles of 0°, 15°, 30°, and 45° to the mesh and use a CFL number that is 0.9 times the maximum shown in either Table 1 or Table 2 or Table 3. We then let the wave number $\left( {k_{x} ,k_{y} } \right)$ sweep through all angles, from $- 180^\circ$ to $+ 180^\circ$ relative to the velocity vector $\left( {{\text{v}}_{x} ,{\text{v}}_{y} } \right)$. For each of those angles, we plot out $1 - \left| {\text{amplification factor}} \right|$ for the scheme. If this number is non-negative and close to zero, it indicates that the scheme has low dissipation. The phase of the amplification factor gives us a measure of the propagation speed of the waves. For each angle between the velocity vector and the wave number, we also plot out the error in the phase speed. As the wavelength increases relative to the zone size, we expect the schemes to propagate waves with increasing accuracy. As a result, we consider wavelengths that are $5 \, \Delta x$, ${10 }\Delta x$, and $15 \, \Delta x$. In the next eight figures that follow, wavelengths of $5 \, \Delta x$ are always shown with a blue color, wavelengths of ${10 }\Delta x$ are always shown with a green color and wavelengths of $15 \, \Delta x$ are always shown with a red color.

**Fig. 5:**
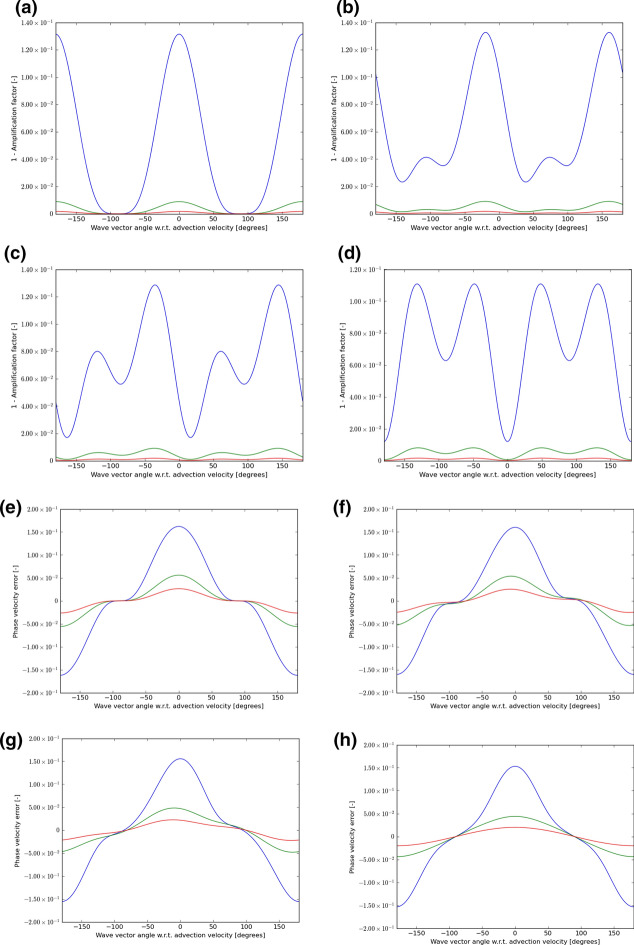
The wave propagation characteristics for curl-preserving second order WENO-like schemes. **a**–**d** one minus the absolute value of the amplification factor when the velocity vector makes angles of 0°, 15°, 30°, and 45° relative to the *x*-direction of the 2D mesh. **e**–**h** the phase error, again for the same angles. The 2D wave vector can make any angle relative to the 2D direction of velocity propagation, therefore, the amplitude and phase information are shown w.r.t. the angle made between the velocity direction and the direction of the wave vector. In each plot, the blue curve refers to waves that span 5 cells per wavelength; the green curve refers to waves that span 10 cells per wavelength; the red curve refers to waves that span 15 waves per wavelength

**Fig. 6:**
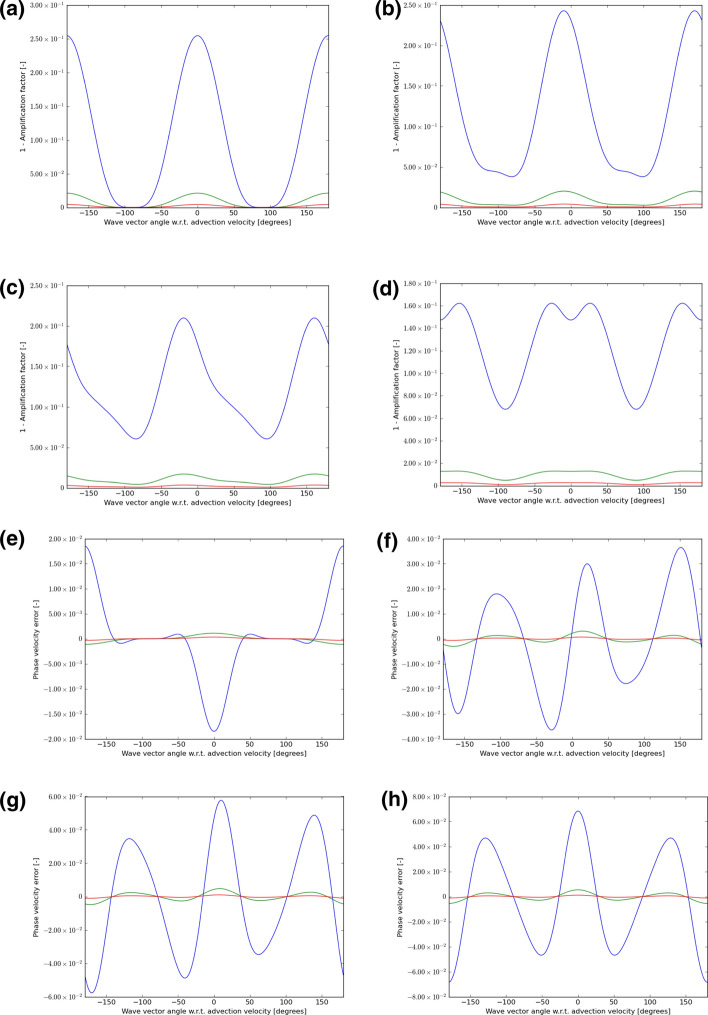
The wave propagation characteristics for curl-preserving third order WENO-like schemes. **a**–**d** one minus the absolute value of the amplification factor when the velocity vector makes angles of 0°, 15°, 30°, and 45° relative to the *x*-direction of the 2D mesh. **e**–**h** the phase error, again for the same angles. The 2D wave vector can make any angle relative to the 2D direction of velocity propagation, therefore, the amplitude and phase information are shown w.r.t. the angle made between the velocity direction and the direction of the wave vector. In each plot, the blue curve refers to waves that span 5 cells per wavelength; the green curve refers to waves that span 10 cells per wavelength; the red curve refers to waves that span 15 waves per wavelength

**Fig. 7:**
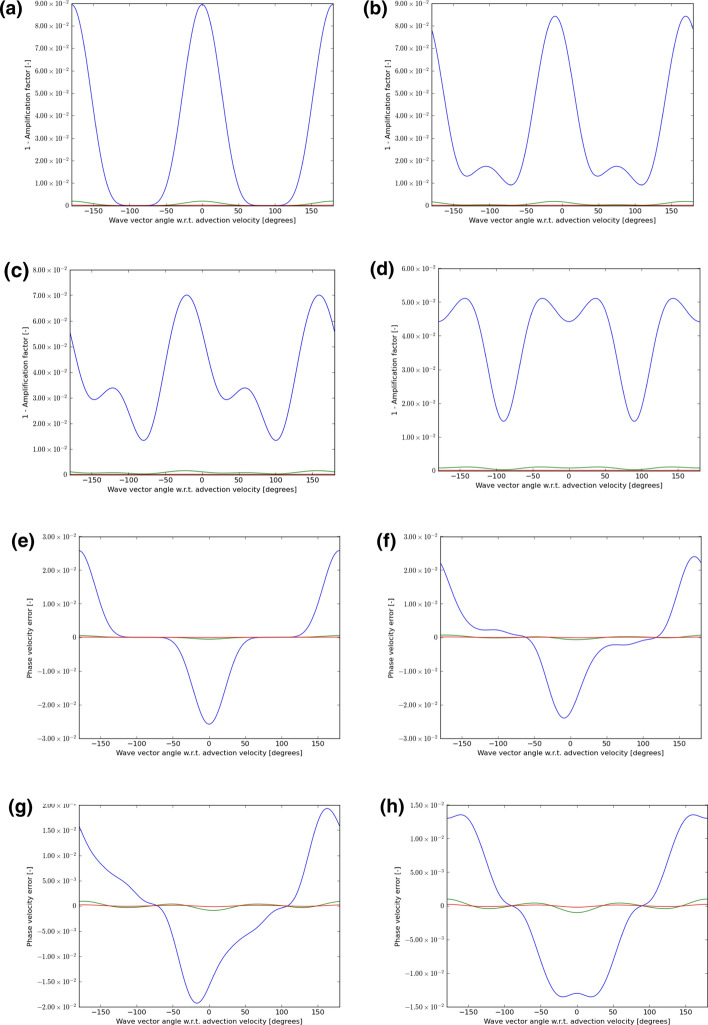
The wave propagation characteristics for curl-preserving fourth order WENO-like schemes. **a**–**d** one minus the absolute value of the amplification factor when the velocity vector makes angles of 0°, 15°, 30° and 45°, relative to the *x*-direction of the 2D mesh. **e**–**h** the phase error, again for the same angles. The 2D wave vector can make any angle relative to the 2D direction of velocity propagation, therefore, the amplitude and phase information are shown w.r.t. the angle made between the velocity direction and the direction of the wave vector. In each plot, the blue curve refers to waves that span 5 cells per wavelength; the green curve refers to waves that span 10 cells per wavelength; the red curve refers to waves that span 15 waves per wavelength

Figures 5, 6, and 7 show the wave propagation characteristics of curl-free WENO-like schemes at second, third and fourth order, respectively. We see that as we go from second to fourth order, the dissipation (as measured by $1 - \left| {\text{amplification factor}} \right|$) improves by an order of magnitude for each of the three wavelengths considered here. Similarly, as we go from second to fourth order, the phase error is also reduced by an order of magnitude. Table 4 shows the minimum of the absolute value of the amplification factor for all possible velocity directions and angles between the velocity and wave number vectors for curl-free WENO-like schemes when we have waves with the wavelength 5Δ*x*, 10Δ*x*, and 15Δ*x*. In the same table, we also show the maximum phase error for the similar situation and for the same wavelengths. In other words, Table 4 was extracted from Figs. 5, 6, and 7 and allows us to quantify the most significant aspect of those figures. Table 4, therefore, allows us to make an important practical decision. Say we want to carry out a simulation with WENO-like schemes we want to meet a target set of dissipation and dispersion properties, Table 4 shows us what our options are. We may indeed choose a lower order scheme and use a lot of zones to cover the characteristic wavelength in the simulation. But we see that we can also choose a higher-order scheme and use fewer zones to cover the characteristic wavelength in the simulation.

**Table 4:** The minimum of the absolute value of the amplification factor for all possible velocity directions and all angles between the velocity and wave number vectors for curl-free WENO-like schemes when we have waves with wavelength 5Δ*x*, 10Δ*x*, and 15Δ*x*

Min of |amplification factor|	*λ* = 5Δ*x*	*λ* = 10Δ*x*	*λ* = 15Δ*x*
WENO-O2	0.867 229 8	0.990 893 0	0.998 172 9
WENO-O3	0.745 507 4	0.978 762 8	0.995 567 1
WENO-O4	0.910 551 6	0.998 038 3	0.999 819 2

Max of phase error	*λ* = 5Δ*x*	*λ* = 10Δ*x*	*λ* = 15Δ*x*
WENO-O2	1.621 195 3E−01	5.597 617 2E−02	2.645 975 3E−02
WENO-O3	6.841 727 1E−02	5.454 241 1E−03	1.145 307 4E−03
WENO-O4	2.581 436 9E−02	1.004 673 7E−03	2.077 598 7E−04
We also show the maximum phase error for the same wavelengths

**Fig. 8:**
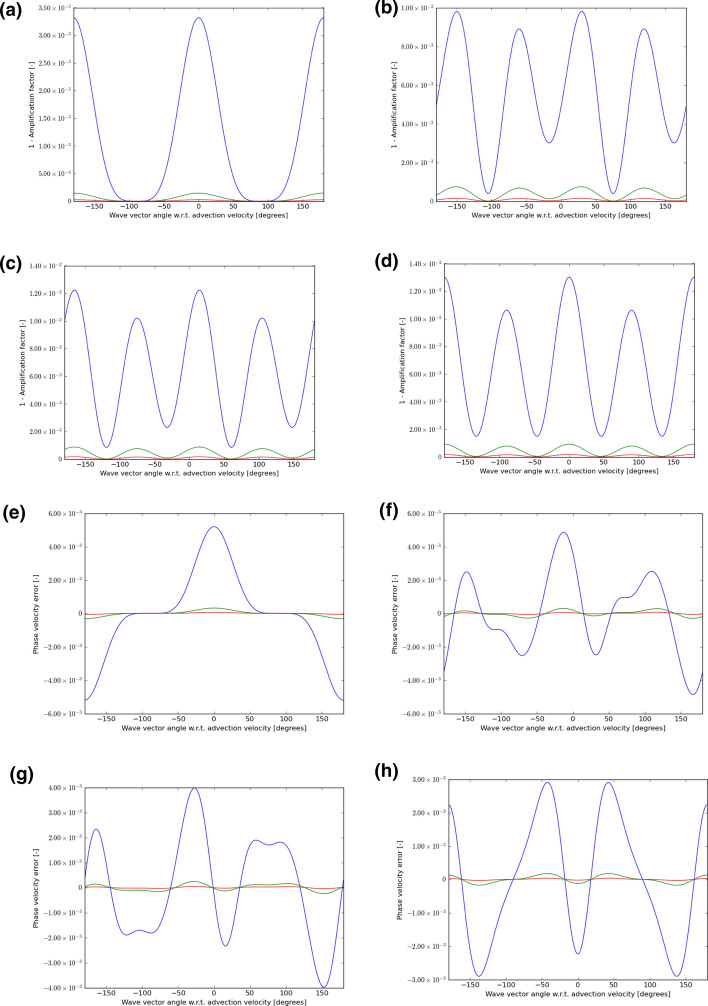
The wave propagation characteristics for curl-preserving third order P1P2-like schemes. **a**–**d** one minus the absolute value of the amplification factor when the velocity vector makes angles of 0°, 15°, 30°, and 45° relative to the *x*-direction of the 2D mesh. **e**–**h** the phase error, again for the same angles. The 2D wave vector can make any angle relative to the 2D direction of velocity propagation, therefore, the amplitude and phase information are shown w.r.t. the angle made between the velocity direction and the direction of the wave vector. In each plot, the blue curve refers to waves that span 5 cells per wavelength; the green curve refers to waves that span 10 cells per wavelength; the red curve refers to waves that span 15 waves per wavelength

**Fig. 9:**
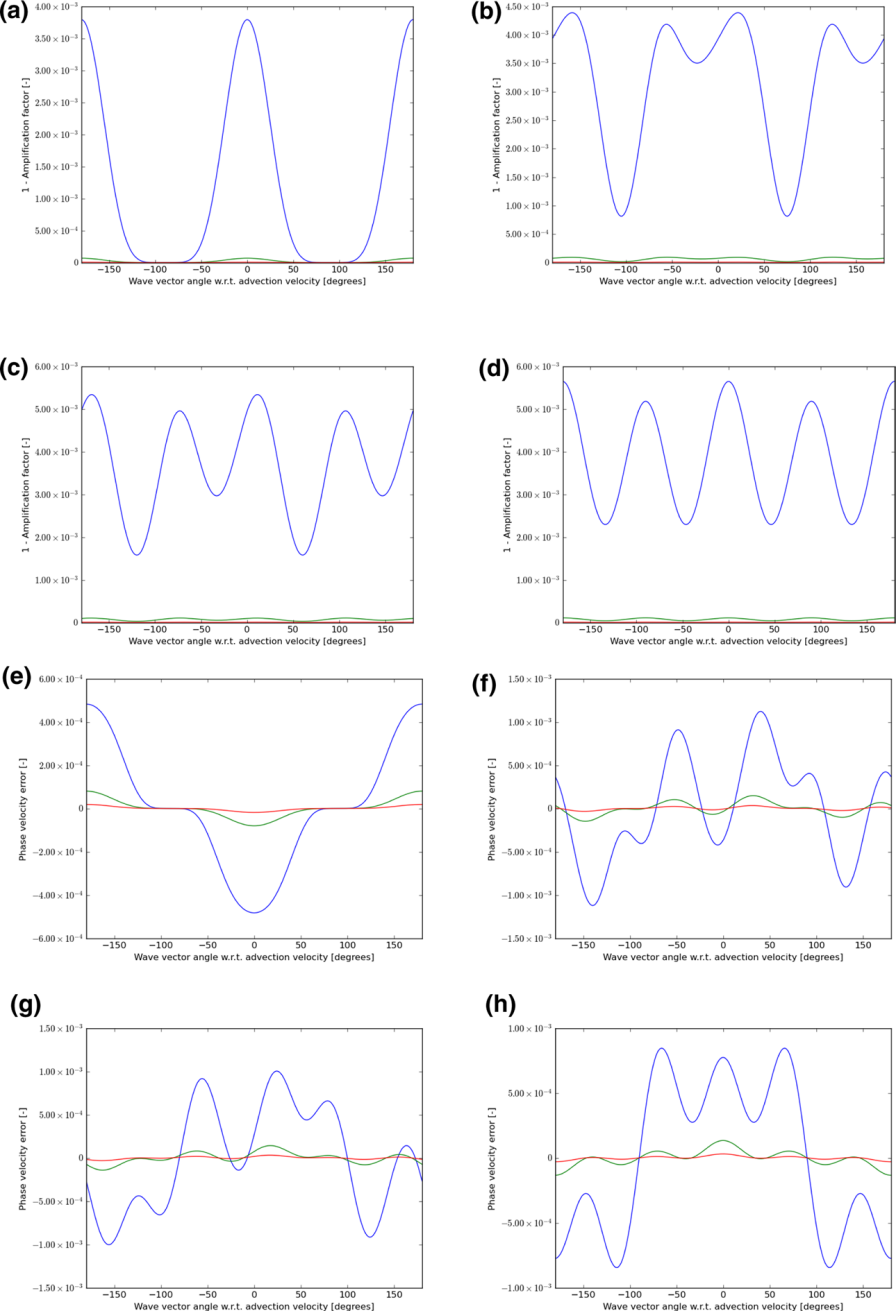
The wave propagation characteristics for curl-preserving fourth order P1P3-like schemes. **a**–**d** one minus the absolute value of the amplification factor when the velocity vector makes angles of 0°, 15°, 30°, and 45° relative to the *x*-direction of the 2D mesh. **e**–**h** the phase error, again for the same angles. The 2D wave vector can make any angle relative to the 2D direction of velocity propagation, therefore, the amplitude and phase information are shown w.r.t. the angle made between the velocity direction and the direction of the wave vector. In each plot, the blue curve refers to waves that span 5 cells per wavelength; the green curve refers to waves that span 10 cells per wavelength; the red curve refers to waves that span 15 waves per wavelength

There is no second-order P1P1 scheme, because such a scheme would be identical to a second-order DG scheme. However, our study of CFL numbers has shown us that curl-free third-order P1P2-like and fourth-order P1P3-like schemes still retain a very robust CFL number. We, therefore, want to know whether such schemes have superior wave propagation characteristics relative to the WENO-like schemes that we studied in the previous paragraph. Figures 8 and 9 show the wave propagation characteristics of curl-free P1P2-like and P1P3-like schemes at third and fourth order, respectively. We should, therefore, compare Fig. 8 to Fig. 6 because they both pertain to third-order schemes. Similarly, we should compare Fig. 9 to Fig. 7 because they both pertain to fourth-order schemes. The results are quite interesting. We see that Fig. 8 and Fig. 6 show comparable quality of wave propagation indicating that at third order the advantages are minimal. This lack of significant improvement might have to do with the fact that SSP-RK3 time stepping has excessive stabilization. Now let us turn to comparing Figs. 9 and 7. At fourth order, we do see that the P1P3-like scheme outperforms the WENO-O4 scheme by almost an order of magnitude. It shows the value of designing P*N*P*M* schemes as half-way houses between WENO and DG schemes. Table 5 shows the minimum of the absolute value of the amplification factor for all possible velocity directions and all angles between the velocity and wave number vectors for curl-free P*N*P*M*-like schemes when we have waves with wavelength 5Δ*x*, 10Δ*x*, and 15Δ*x*. In the same table, we also show the maximum phase error for a similar situation and for the same wavelengths. In other words, Table 5 was extracted from Figs. 8 and 9 and allows us to quantify the most significant aspect of those figures. Again, Table 5 can help with practical decision-making. It shows us, for example, that fourth-order P1P3-like schemes do give us a substantial improvement over fourth-order WENO-like scheme while incurring only a modest increase in computational complexity.

**Table 5:** The minimum of the absolute value of the amplification factor for all possible velocity directions and all angles between the velocity and wave number vectors for curl-free P*N*P*M*-like schemes for waves with wavelength 5Δ*x*, 10Δ*x*, and 15Δ*x*

Min of |amplification factor|	*λ* = 5Δ*x*	*λ* = 10Δ*x*	*λ* = 15Δ*x*
P1P2	0.986 983 0	0.999 072 2	0.999 811 8
P1P3	0.994 354 9	0.999 891 3	0.999 989 8

Max of phase error	*λ* = 5Δ*x*	*λ* = 10Δ*x*	*λ* = 15Δ*x*
P1P2	5.200 135 1E−03	3.197 237 9E−04	6.493 185 6E−05
P1P3	1.122 064 2E−03	1.477 350 8E−04	3.295 251 5E−05

We also show the maximum phase error for the same wavelengths

**Fig. 10:**
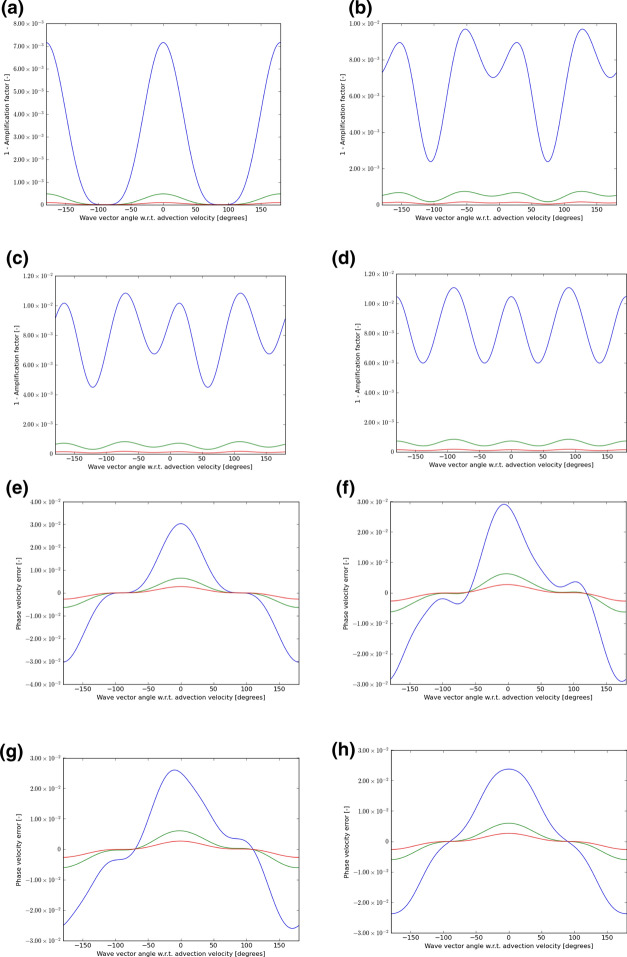
The wave propagation characteristics for curl-preserving second order DG-like schemes. **a**–**d** one minus the absolute value of the amplification factor when the velocity vector makes angles of 0°, 15°, 30°, and 45°, relative to the *x*-direction of the 2D mesh. **e**–**h** the phase error, again for the same angles. The 2D wave vector can make any angle relative to the 2D direction of velocity propagation, therefore, the amplitude and phase information are shown w.r.t. the angle made between the velocity direction and the direction of the wave vector. In each plot, the blue curve refers to waves that span 5 cells per wavelength; the green curve refers to waves that span 10 cells per wavelength; the red curve refers to waves that span 15 waves per wavelength

**Fig. 11:**
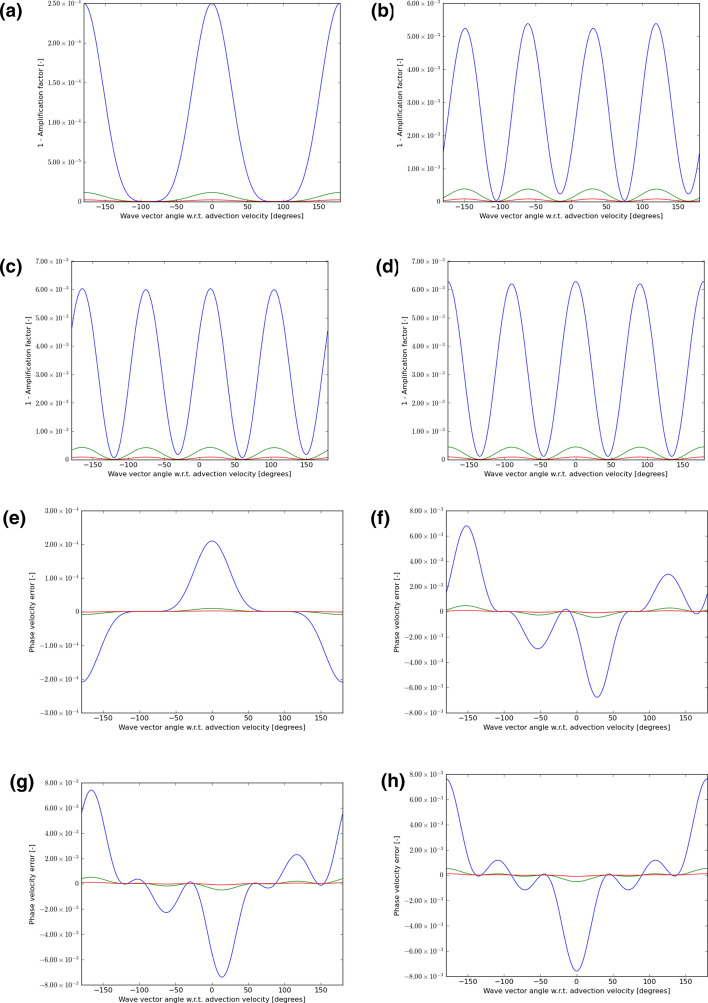
The wave propagation characteristics for curl-preserving third order DG-like schemes. **a**–**d** one minus the absolute value of the amplification factor when the velocity vector makes angles of 0°, 15°, 30°, and 45° relative to the *x*-direction of the 2D mesh. **e**–**h** the phase error, again for the same angles. The 2D wave vector can make any angle relative to the 2D direction of velocity propagation, therefore, the amplitude and phase information are shown w.r.t. the angle made between the velocity direction and the direction of the wave vector. In each plot, the blue curve refers to waves that span 5 cells per wavelength; the green curve refers to waves that span 10 cells per wavelength; the red curve refers to waves that span 15 waves per wavelength

**Fig. 12:**
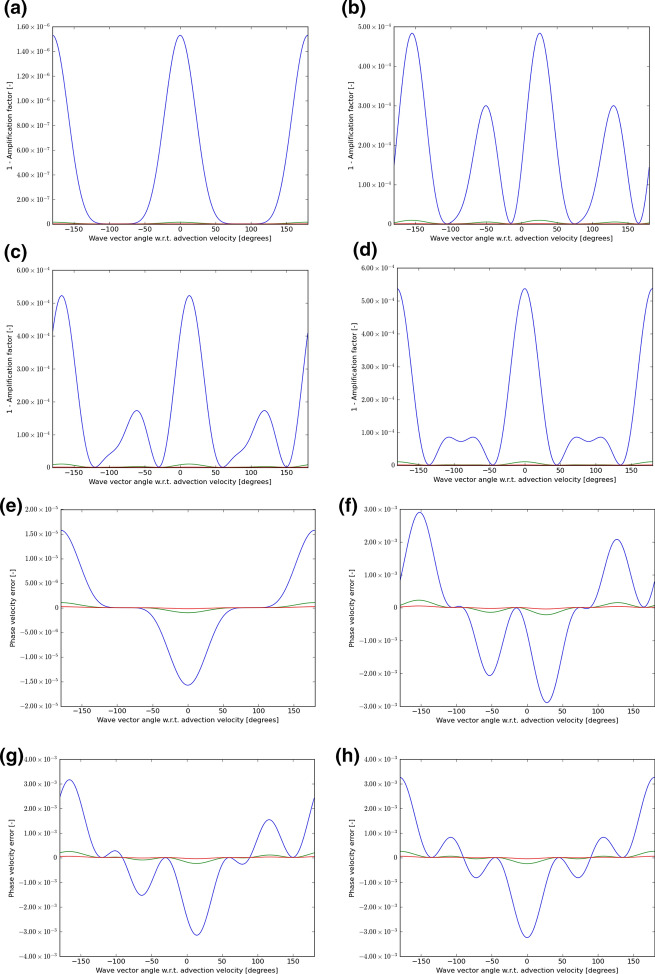
The wave propagation characteristics for curl-preserving fourth order DG-like schemes. **a**–**d** one minus the absolute value of the amplification factor when the velocity vector makes angles of 0°, 15°, 30°, and 45° relative to the *x*-direction of the 2D mesh. **e**–**h** the phase error, again for the same angles. The 2D wave vector can make any angle relative to the 2D direction of velocity propagation, therefore, the amplitude and phase information are shown w.r.t. the angle made between the velocity direction and the direction of the wave vector. In each plot, the blue curve refers to waves that span 5 cells per wavelength; the green curve refers to waves that span 10 cells per wavelength; the red curve refers to waves that span 15 waves per wavelength

While they have the smallest CFL numbers, DG-like schemes hold out the promise of almost spectral-like accuracy with increasing order of accuracy; for finite volume-based approaches this is now viewed as an accepted fact. We are now in a position to test that contention as it pertains to curl-free DG-like schemes. Figures 10, 11, and 12 show the wave propagation characteristics of curl-free DG-like schemes at second, third, and fourth order, respectively. With increasing order, the curl-free DG-like schemes do show significant improvement when inter-compared amongst themselves. Let us, therefore, compare across algorithms since we have all the data concatenated in one place. Figure 10 should be compared to Fig. 5. Figure 11 should be compared to Figs. 6 and 8. Figure 12 should be compared to Figs. 7 and 9. We see that the wave propagation characteristics of the second-order DG-like scheme are entirely competitive with the wave propagation characteristics of the fourth-order WENO-like scheme. The fourth-order DG-like scheme is also somewhat superior to the fourth-order P1P3-like scheme, but please recall that this comes with a substantial increase in programming complexity and a decrease in the CFL number. Table 6 shows the minimum of the absolute value of the amplification factor for all possible velocity directions and all angles between the velocity and wave number vectors for curl-free DG-like schemes when we have waves with wavelength 5Δ*x*, 10Δ*x*, and 15Δ*x*. In the same table, we also show the maximum phase error for a similar situation and for the same wavelengths. In other words, Table 6 was extracted from Figs. 10, 11, and 12 and allows us to quantify the most significant aspect of those figures. We see that the dissipation and dispersion characteristics of the curl-free DG-like schemes that we have designed are indeed excellent. Comparing Tables 5 and 6 we also see that the curl-free P*N*P*M*-like schemes are not far behind. Therefore, DG-like schemes are the go-to scheme when superlative performance is the only driving consideration. However, if one wants lower computational complexity and more robust timesteps, the P*N*P*M*-like schemes also present themselves as attractive choices.

**Table 6:** The minimum of the absolute value of the amplification factor for all possible velocity directions and all angles between the velocity and wave number vectors for curl-free DG-like schemes when we have waves with wavelength 5Δ*x*, 10Δ*x*, and 15Δ*x*

Min of |amplification factor|	*λ* = 5Δ*x*	*λ* = 10Δ*x*	*λ* = 15Δ*x*
DG *P* = 1	0.988 938 3	0.999 153 4	0.999 825 1
DG *P* = 2	0.993 718 9	0.999 556 5	0.999 910 5
DG *P* = 3	0.999 463 3	0.999 989 7	0.999 999 1

Max of phase error	*λ* = 5Δ*x*	*λ* = 10Δ*x*	*λ* = 15Δ*x*
DG *P* = 1	3.034 481 3E−02	6.420 087 7E−03	2.737 861 6E−03
DG *P* = 2	7.607 727 1E−03	5.194 247 2E−04	1.041 523 8E−04
DG *P* = 3	3.254 652 1E−03	2.512 749 9E−04	5.180 446 8E−05

We also show the maximum phase error for the same wavelengths

While this section has been focused on curl-free methods, we point out that curl-preserving methods only require a few additional terms in Eqs. (10) and (11) compared to curl-free schemes. Without an underlying fluid-dynamical PDE system that supplies additional terms like density, velocity, and temperature, it is not possible to obtain the source terms and other types of terms which would make Eq. (2) curl-preserving instead of curl-free. Furthermore, computer algebra systems are just not adept enough to support a more extensive stability analysis for larger PDE systems. For these reasons, the analysis presented here is focused on curl-free methods, but the insights developed here will extend to all curl-preserving methods.

## Numerical Results

In this section, we present numerical experiments confirming that the developed curl-free DG schemes reach their expected design accuracies. Results for all the DG-like (P*N*P*N*) and P*N*P*M* (P0P*N* for WENO-like and P1P*N* for HWENO-like) up to fourth order are reported for two smooth test problems. All the tests are run with 95% of the maximal CFL number (Tables 1, 2, 3) of the respective scheme.

### Plane-Wave Test Problem

The first test problem is the propagation of a plane wave in a Cartesian domain of size $\left[ { - 1/2,  1/2} \right]^{2}$ with periodic boundaries. The plane wave is advected with velocity ${\text{v}}_{x} = {\text{v}}_{y} = 1$ diagonally through the domain. The curl-free field ${\mathbf{J}}$ is initialized from a potential

$$ \phi (x,y) = \cos (k_{x} x + k_{y} y), $$

where we set $$k_{x} = k_{y} = $$2π. The *x*- and *y*-field components of the curl-free field are then given by

$$ J_{x} = \frac{\partial \phi }{{\partial x}}\quad {\text{and}}\quad J_{y} = \frac{\partial \phi }{{\partial y}}. $$

The setup is run up to time $t_{f} = 1$, by which time the plane wave has propagated once through the domain, and the accuracy of the schemes is evaluated. Moreover, we also show the preservation of the quadratic field energy $(J_{x}^{2} + J_{y}^{2} )/2$ highlighting the dissipation characteristics of the schemes.

Table 7 shows the $L_{1}$ and $L_{\infty }$ errors at the final time for the P*N*P*N* (*N* = 1, 2, 3) DG-like schemes for resolutions from 8 × 8 up to 64 × 64. Table 8 shows the convergence analysis of the P0P*N* (*N* = 1, 2, 3) WENO-like schemes. Table 9 shows the convergence analysis of the P1P*N* (*N* = 1, 2, 3) HWENO-like schemes. The tables also catalogue the final quadratic energy as a fraction of the initial quadratic energy. We observe that all schemes reach their design accuracy. We also take note of the improved quadratic field energy preservation with increasing order of accuracy. Note that we did nothing special in the scheme to ensure that quadratic field energy is conserved; as a result, the rather good preservation of quadratic energy is entirely a consequence of the accuracy of the method. This is especially true for the DG-like schemes which preserve quadratic energy very well especially as the resolution is increased. The WENO-like schemes show slightly inferior energy preservation characteristics. However, the latter allow much larger time steps due to their larger allowed CFL numbers. The HWENO-like schemes show nearly the same quadratic energy preservation properties as the DG-like schemes and, furthermore, allow larger time steps similar to the WENO-like schemes. Consequently, we see that the HWENO-like schemes may be viewed as an efficient compromise between the extreme accuracy of the DG-like schemes and the much larger time steps of the WENO-like schemes.

**Table 7:** Accuracy analysis (plane wave test) of the P*N*P*N* (*N* = 1,2,3) DG-like schemes

P1P1	*L*_1_ error	*L*_1_ accuracy	*L*_∞_ error	*L*_∞_ accuracy	Total quadratic energy
8 × 8	1.054E+00	–	1.710E+00	–	0.767 072 144 659 713
16 × 16	1.959E−01	2.43	3.041E−01	2.49	0.963 508 927 621 496
32 × 32	3.642E−02	2.43	5.699E−02	2.42	0.995 169 598 723 023
64 × 64	7.897E−03	2.21	1.240E−02	2.20	0.999 386 133 084 480

P2P2	*L*_1_ error	*L*_1_ accuracy	*L*_∞_ error	*L*_∞_ accuracy	Total quadratic energy
8 × 8	8.529E−01	–	1.335E+00	–	0.798 900 332 986 684
16 × 16	1.229E−01	2.79	1.931E−01	2.79	0.969 506 454 484 418
32 × 32	1.584E−02	2.96	2.488E−02	2.96	0.996 044 633 506 995
64 × 64	1.993E−03	2.99	3.130E−03	2.99	0.999 501 861 659 339

P3P3	*L*_1_ error	*L*_1_ accuracy	*L*_∞_ error	*L*_∞_ accuracy	Total quadratic energy
8 × 8	1.150E−01	–	1.711E−01	–	0.982 433 477 747 556
16 × 16	8.007E−03	3.84	1.235E−02	3.79	0.999 160 570 223 597
32 × 32	5.131E−04	3.96	8.082E−04	3.93	0.999 961 937 114 448
64 × 64	3.256E−05	3.98	5.115E−05	3.98	0.999 998 147 852 102

The total quadratic energy on the mesh as a fraction of its initial value is also shown

**Table 8:** Accuracy analysis (plane wave test) of the P0P*N* (*N* = 1,2,3) WENO-like schemes

P0P1	*L*_1_ error	*L*_1_ accuracy	*L*_∞_ error	*L*_∞_ accuracy	Total quadratic energy
8 × 8	4.993E+00	–	6.970E+00	–	0.147 331 805 631 007
16 × 16	1.687E+00	1.57	3.081E+00	1.18	0.672 786 308 056 742
32 × 32	7.354E−01	1.20	1.342E+00	1.20	0.961 889 800 888 593
64 × 64	1.939E−01	1.92	5.060E−01	1.41	0.996 184 224 345 619

P0P2	*L*_1_ error	*L*_1_ accuracy	*L*_∞_ error	*L*_∞_ accuracy	Total quadratic energy
8 × 8	2.377E+00	–	3.458E+00	–	0.493 454 736 716 243
16 × 16	3.817E−01	2.64	5.868E−01	2.56	0.906 990 382 419 879
32 × 32	5.000E−02	2.93	7.805E−02	2.91	0.987 543 393 334 568
64 × 64	6.291E−03	2.99	9.866E−03	2.98	0.998 428 021 784 668

P0P3	*L*_1_ error	*L*_1_ accuracy	*L*_∞_ error	*L*_∞_ accuracy	Total quadratic energy
8 × 8	5.523E−01	–	9.562E−01	–	0.863 259 629 337 563
16 × 16	1.244E−02	5.47	3.046E−02	4.97	0.996 553 938 792 429
32 × 32	3.951E−04	4.98	8.497E−04	5.16	0.999 902 248 490 279
64 × 64	1.387E−05	4.83	2.503E−05	5.09	0.999 997 008 920 715

The total quadratic energy on the mesh as a fraction of its initial value is also shown

**Table 9:** Accuracy analysis (plane wave test) of the P1P*N* (*N* = 2, 3) HWENO-like schemes

P1P2	*L*_1_ error	*L*_1_ accuracy	*L*_∞_ error	*L*_∞_ accuracy	Total quadratic energy
8 × 8	8.478E−01	–	1.254E+00	–	0.800 179 565 838 325
16 × 16	1.244E−01	2.77	1.918E−01	2.71	0.969 170 286 097 292
32 × 32	1.628E−02	2.93	2.546E−02	2.91	0.995 933 787 552 492
64 × 64	2.065E−03	2.98	3.239E−03	2.97	0.999 483 905 512 296

P1P3	*L*_1_ error	*L*_1_ accuracy	*L*_∞_ error	*L*_∞_ accuracy	Total quadratic energy
8 × 8	2.331E−01	–	3.870E−01	–	0.942 799 109 295 127
16 × 16	1.341E−02	4.12	2.090E−02	4.21	0.997 772 917 919 100
32 × 32	7.612E−04	4.14	1.183E−03	4.14	0.999 927 383 586 396
64 × 64	4.600E−05	4.05	7.202E−05	4.04	0.999 997 714 221 129

The total quadratic energy on the mesh as a fraction of its initial value is also shown

### Vortex Test Problem

The second test problem consists of the advection of a localized curl-free vortex similar to the magnetic vortex for the induction equation. The Cartesian domain extents are $[ - 10, 10]^{2}$ with periodic boundary condition. The vortex is initialized from a potential

$$ \phi (x,y) = {\text{e}}^{{\frac{1}{2}(1 - r^{2} )}} , $$

where $r = \sqrt {x^{2} + y^{2} }$. This results in a field given by

$$ {\mathbf{J}} = \nabla \phi = - {\text{e}}^{{\frac{1}{2}(1 - r^{2} )}} [x,y]^{{\text{T}}} . $$

The advection velocity is set to ${\text{v}}_{x} = {\text{v}}_{y} = 1$. The problem is simulated for the time $t_{f} = 20$, by which point the vortex was advected once through the square domain in the diagonal direction till it returns to its initial position. At final time point, we measure the accuracy in the $L_{1}$ and $L_{\infty }$ errors norms. Moreover, we also show the preservation of the quadratic field energy $(J_{x}^{2} + J_{y}^{2} )/2$ highlighting the dissipation characteristics of the schemes.

Table 10 shows the $L_{1}$ and $L_{\infty }$ errors at the final time for the P*N*P*N* (*N* = 1, 2, 3) RKDG-like schemes for resolutions from 16 × 16 up to 256 × 256. Table 11 shows the convergence analysis of the P0P*N* (*N* = 1, 2, 3) WENO-like schemes. Table 12 shows the convergence analysis of the P1P*N* (*N* = 1, 2, 3) HWENO-like schemes. The tables also catalogue the final quadratic energy as a fraction of the initial quadratic energy. We observe that all schemes reach their design accuracy on the chosen mesh resolutions, even for this highly spatially localized vortex. Note that much of the field variation is confined around a circle with unit radius, corresponding to one-tenth of the computational domain. We find that the presented schemes concurrently have quadratic energy preservation with, nevertheless, excellent accuracy.

**Table 10:** Accuracy analysis (vortex test) of the P*N*P*N* (*N* = 1, 2, 3) DG-like schemes

P1P1	*L*_1_ error	*L*_1_ accuracy	*L*_∞_ error	*L*_∞_ accuracy	Total quadratic energy
16 × 16	3.960E−02	–	1.296E+00	–	0.244 138 062 854 683
32 × 32	1.937E−02	1.03	9.775E−01	0.41	0.584 068 951 760 809
64 × 64	4.780E−03	2.02	3.237E−01	1.59	0.887 813 286 147 527
128 × 128	8.569E−04	2.48	6.715E−02	2.27	0.982 478 078 399 363
256 × 256	1.678E−04	2.35	1.243E−02	2.43	0.997 712 794 953 464

P2P2	*L*_1_ error	*L*_1_ accuracy	*L*_∞_ error	*L*_∞_ accuracy	Total quadratic energy
16 × 16	3.813E−02	–	1.017E+00	–	0.449 340 807 458 768
32 × 32	1.535E−02	1.31	6.368E−01	0.67	0.743 031 482 000 765
64 × 64	3.249E−03	2.24	1.748E−01	1.87	0.938 198 337 740 548
128 × 128	4.658E−04	2.80	2.755E−02	2.67	0.990 782 650 850 455
256 × 256	5.981E−05	2.96	3.601E−03	2.94	0.998 809 736 688 618

P3P3	*L*_1_ error	*L*_1_ accuracy	*L*_∞_ error	*L*_∞_ accuracy	Total quadratic energy
16 × 16	1.672E−02	–	5.812E−01	–	0.837 642 154 450 034
32 × 32	2.898E−03	2.53	1.281E−01	2.18	0.980 766 190 163 135
64 × 64	2.427E−04	3.58	1.200E−02	3.42	0.999 051 579 122 300
128 × 128	1.607E−05	3.92	8.047E−04	3.90	0.999 964 537 681 624
256 × 256	1.019E−06	3.98	5.100E−05	3.98	0.999 998 674 687 398

The total quadratic energy on the mesh as a fraction of its initial value is also shown

**Table 11:** Accuracy analysis (vortex test) of the P0P*N* (*N* = 1, 2, 3) WENO-like schemes

P0P1	*L*_1_ error	*L*_1_ accuracy	*L*_∞_ error	*L*_∞_ accuracy	Total quadratic energy
16 × 16	4.396E−02	–	1.408E+00	–	0.016 052 344 223 764
32 × 32	3.955E−02	0.15	1.771E+00	− 0.33	0.060 627 418 657 843
64 × 64	2.399E−02	0.72	1.357E+00	0.38	0.274 789 236 132 377
128 × 128	7.655E−03	1.65	5.670E−01	1.26	0.773 714 109 741 705
256 × 256	1.988E−03	1.94	1.543E−01	1.88	0.979 590 328 058 657

P0P2	*L*_1_ error	*L*_1_ accuracy	*L*_∞_ error	*L*_∞_ accuracy	Total quadratic energy
16 × 16	4.036E−02	–	1.418E+00	–	0.014 812 755 301 855
32 × 32	3.251E−02	0.31	1.671E+00	− 0.24	0.113 246 493 972 690
64 × 64	1.018E−02	1.67	6.669E−01	1.32	0.692 818 242 056 277
128 × 128	1.995E−03	2.35	1.484E−01	2.17	0.942 819 902 537 954
256 × 256	2.689E−04	2.89	2.108E−02	2.82	0.992 210 030 651 288

P0P3	*L*_1_ error	*L*_1_ accuracy	*L*_∞_ error	*L*_∞_ accuracy	Total quadratic energy
16 × 16	3.903E−02	–	1.409E+00	–	0.042 648 512 931 034
32 × 32	1.931E−02	1.02	1.087E+00	0.37	0.483 958 370 120 984
64 × 64	1.940E−03	3.31	1.397E−01	2.96	0.953 798 557 685 217
128 × 128	8.435E−05	4.52	5.971E−03	4.55	0.998 754 807 116 245
256 × 256	4.283E−06	4.30	2.492E−04	4.58	0.999 964 258 479 139

The total quadratic energy on the mesh as a fraction of its initial value is also shown

**Table 12:** Accuracy analysis (vortex test) of the P1P*N* (*N* = 2, 3) HWENO-like schemes

P1P2	*L*_1_ error	*L*_1_ accuracy	*L*_∞_ error	*L*_∞_ accuracy	Total quadratic energy
16 × 16	3.629E−02	–	1.172E+00	–	0.288 607 282 457 506
32 × 32	1.563E−02	1.21	7.317E−01	0.68	0.675 080 995 909 535
64 × 64	3.281E−03	2.25	1.797E−01	2.03	0.932 780 213 745 329
128 × 128	4.730E−04	2.79	2.774E−02	2.70	0.990 441 076 783 871
256 × 256	6.101E−05	2.95	3.655E−03	2.92	0.998 771 733 291 638

P1P3	*L*_1_ error	*L*_1_ accuracy	*L*_∞_ error	*L*_∞_ accuracy	Total quadratic energy
16 × 16	2.919E−02	–	1.055E+00	–	0.346 983 109 358 981
32 × 32	7.340E−03	1.99	3.727E−01	1.50	0.872 263 471 054 451
64 × 64	5.285E−04	3.80	3.912E−02	3.25	0.992 401 678 126 276
128 × 128	2.700E−05	4.29	1.909E−03	4.36	0.999 731 098 030 406
256 × 256	1.514E−06	4.16	9.190E−05	4.38	0.999 991 386 714 680

The total quadratic energy on the mesh as a fraction of its initial value is also shown

**Fig. 13:**
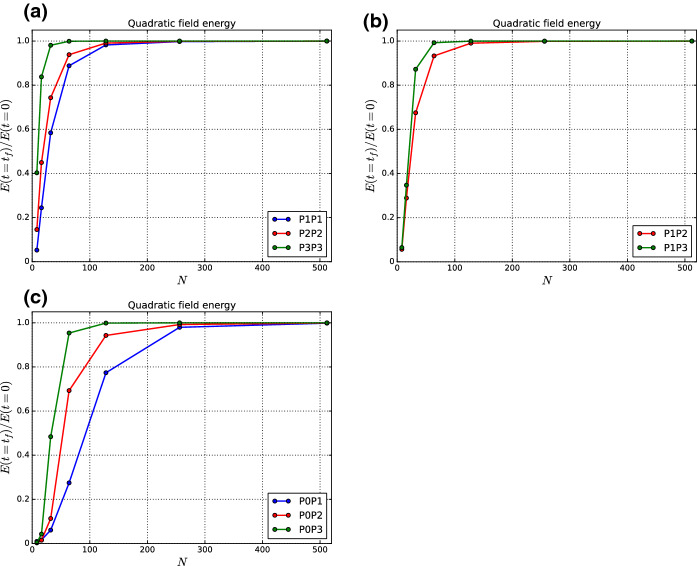
The quadratic field energy from the vortex problem preserved on the mesh at the final time point in the simulation as a function of mesh size. **a** the curl-free DG-like schemes, **b** the curl-free P1P*N*-like schemes, and **c** the curl-free WENO-like schemes

As before, we did nothing special in the scheme to ensure that the quadratic field energy is conserved. Consequently, the rather good preservation of quadratic energy is entirely a consequence of the accuracy of the method. For the quadratic field energy, we observe similar trends to the plane wave test problem. To further highlight the point, Fig. 13 shows the quadratic field energy preservation characteristics from Tables 10, 11, and 12, graphically. Each panel shows all the available schemes up to fourth order of accuracy. Figure 13a underlines that the curl-free DG-like schemes show excellent energy-preserving properties with increasing order of accuracy. In Fig. 13b, we observe again that the energy preserving properties of the curl-free WENO-like schemes are somewhat inferior in the pre-asymptotic regime. However, the improving trend with increasing order of accuracy is also clearly visible. In Fig. 13c, we see that the curl-free P1P*N*-like schemes share almost the same preservation properties as the curl-free DG-like schemes. The latter fact, and their substantially larger allowed CFL numbers (hence, time steps), highlight again that the P1P*N*-like schemes are very efficient curl constraint-preserving methods that share desirable qualities from both full DG-like and WENO-like schemes.

Lastly, we also catalogue that the schemes designed here are curl-preserving and can indeed reach the curl-free limit even when they are integrated for long simulation times. Dumbser et al. [28] have shown that if a classical higher-order Godunov scheme is applied to Eq. (2), the numerical instability manifests itself as an explosive increase in the discrete circulation when the simulation is run over long periods of time; see [28, Fig. 5]. Therefore, we would like to demonstrate that the discrete circulation is held down to machine precision when the simulation is run for a long period of time when we use the methods designed here. We would indeed like to go one step further and plot out the time series of the maximum pointwise error in the curl of **J**. In other words, we know that the vector field is a polynomial, see Eq. (14) or Eq. (17) for instance, so that we can evaluate the pointwise curl at any point within a zone (because the polynomials are differentiable). We then choose 2 × 2, 3 × 3, or 4 × 4 uniformly spaced points that are internal to each zone at second, third, and fourth orders, respectively. We then evaluate the maximum of the absolute value of the curl at each and every internal point for all the zones on the mesh and we plot this maximum value as a function of time. Let us ask why this demonstration matters? We see from Eqs. (10) and (11) that in a curl-preserving scheme we will also need terms like $\left\langle {{\text{v}}_{x} \left( {\nabla \times {\mathbf{J}}} \right)_{z} } \right\rangle$ and $\left\langle {{\text{v}}_{y} \left( {\nabla \times {\mathbf{J}}} \right)_{z} } \right\rangle$, and other terms like it, at the edges of the mesh. These have to be evaluated from either side of the edge that is being considered. Therefore, when we approach the curl-free limit, a curl-preserving scheme should naturally obtain curl-free evolution. This demonstration that the maximum pointwise error in the curl of **J** remains close to machine zero over long simulation times guarantees that such a limit is met.

**Fig. 14:**
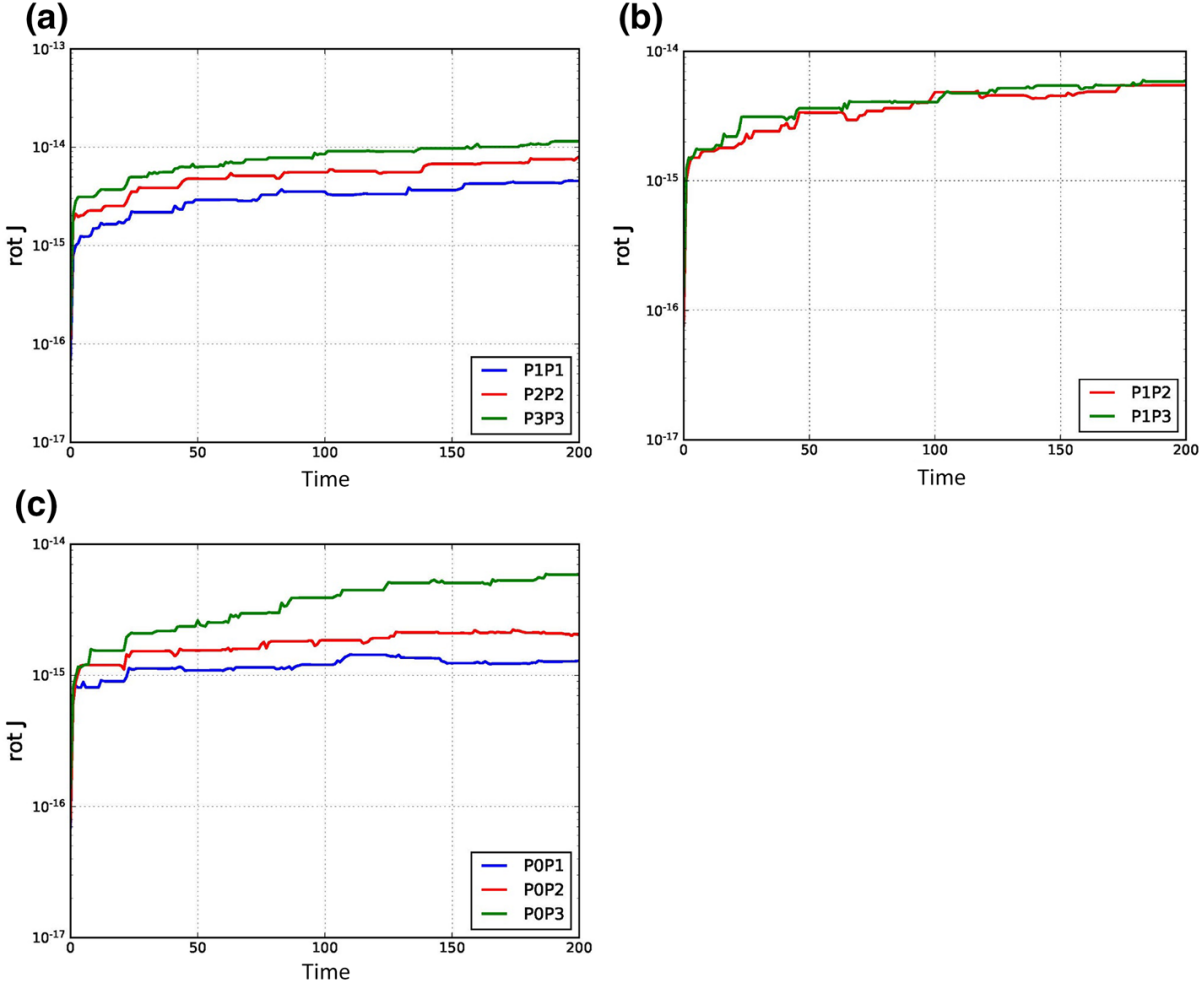
The maximum pointwise error of the curl of **J** as a function of time for a 64 × 64 zone run of the vortex problem. **a** the evolution of the maximum pointwise curl as a function of time for the second, third, and fourth order curl-free DG-like schemes. **b** the evolution of the maximum pointwise curl as a function of time for the third and fourth order curl-free P1PN-like schemes. **c** the evolution of the maximum pointwise curl as a function of time for the second, third and fourth order curl-free WENO-like schemes. The figure shows that all our curl-preserving schemes can preserve the curl constraint up to machine accuracy

To show that the curl remains close to machine zero at all points on the mesh even during long-time integration, we have run the vortex problem on a 64 × 64 zone mesh to a final time of 200. This time corresponds to the vortex making ten passages through the periodic computational domain. Figure 14 shows the maximum pointwise error of the curl of **J** as a function of time for a 64 × 64 zone run of the vortex problem. Figure 14a shows the evolution of the maximum pointwise curl as a function of time for the second, third, and fourth order curl-free DG-like schemes. Figure 14b shows the evolution of the maximum pointwise curl as a function of time for the third and fourth order curl-free P1P*N*-like schemes. Figure 14c shows the evolution of the maximum pointwise curl as a function of time for the second, third, and fourth order curl-free WENO-like schemes. The figure shows that all our curl-preserving schemes can preserve the curl constraint up to machine accuracy in simulations that are run for long integration times.

## Conclusions

Novel classes of PDEs have recently emerged and the physics of the PDEs requires keeping strict control of the curl of one or more vector fields. The PDEs are hyperbolic systems of great interest to science and engineering. Many of the hyperbolic systems resulting from the GPR formulation for hyperelasticity and compressible multiphase flow with and without surface tension have curl-preserving update equations (Godunov and Romenski [32], Romenski [40], Romenski et al. [41], Peshkov and Romenski [37, 38], Dumbser et al. [27, 30, 31], Schmidmayer et al. [42]). The equations of general relativity, when cast in the FO-CCZ4 formulation, also have such a structure (Alic et al. [1, 2], Brown et al. [18], Dumbser et al. [28, 29]). Similarly, it has recently become possible to recast Schrödinger’s equation in first-order hyperbolic form, and the time-evolution of this important equation also has curl-preserving constraints (Dhaouadi et al. [25], Busto et al. [19]). Experience has shown that if nothing special is done to account for the curl-preserving vector field, it can blow up in a finite amount of simulation time (Dumbser et al. [28]).

Prior work has shown that classical zone-centered Godunov methods can be adapted to such systems only if a GLM-type cleaning approach is included to suppress the build up of circulation on the mesh (Dumbser et al. [28]). The two-fold problem with this approach is as follows: (i) we often get a very large system of Lagrange multipliers that are not part of the original PDE system and (ii) the signal speed with which the Lagrange multipliers have to be advected often exceeds the physical signal speed in the problem by a substantial margin. Another alternative is to solve an elliptical system at every timestep (Boscheri et al. [17]), which makes each timestep very expensive. In Balsara et al. [12] we first presented curl-preserving WENO-like methods that overcame both of the above-mentioned limitations. The methods were based on inventing a novel globally curl-preserving reconstruction strategy that reconstructs the vector field over the zone’s volume using the components of the vector field that were collocated at the edges of the mesh. Non-linear hybridization, via WENO methods, was seamlessly built into the curl-preserving reconstruction strategy. These edge-centered components were updated using multidimensionally upwinded potentials that were collocated at the vertices of the mesh. Multidimensional Riemann solvers, designed by the first author, provided the requisite multidimensional upwinding. The resulting highly stable finite volume-like schemes for curl-preserving systems had the following desirable properties. (i) They did not blow up even after very long integration times. (ii) They did not need GLM-style cleaning with very high signal speeds. (iii) They could operate with large explicit timesteps. (iv) They did not require the solution of an elliptic system. And (v) they could be extended to higher orders while incorporating non-linear hybridization using WENO-like methods. It is, therefore, desirable to invent DG-like and P*N*P*M*-like variants of these WENO-like schemes so that they can inherit the same desirable features—such a task is fulfilled in this paper.

Since we know that DG and P*N*P*M* schemes provide more accurate alternatives to WENO schemes, it becomes interesting to design curl-free and curl-preserving variants of the such schemes. In this paper, we present for the very first time, globally curl-preserving DG-like and P*N*P*M*-like schemes that share the beneficial traits of the globally curl-preserving WENO-like schemes designed by Balsara et al. [12]. The higher moments of the vector components that live in the edges of the mesh are, therefore, endowed with time-evolution that is consistent with the governing equations. This is accomplished by making a Galerkin projection within each edge that results in a weak form of update equation for the higher-order edge-centered moments. The update utilizes the multidimensionally upwinded potentials at the vertices of the mesh. Such update equations have been documented in Sect. 2 for the model Eq. (2) and some nuances of the curl-preserving reconstruction, and how it relates to traditional DG schemes, are highlighted in Sect. 3.

It is well-known that zone-centered DG schemes have wave propagation characteristics superior to zone-centered WENO schemes. Such a superior behavior can be revealed by conducting a von-Neumann stability analysis of either scheme and inter-comparing the results. It is, therefore, interesting to carry out a von Neumann stability analysis of our newly-developed globally curl-free and curl-preserving DG-like and P*N*P*M*-like schemes. In Sect. 4 we present details of our von Neumann stability analysis. To highlight the curl-free aspect of the evolution, the analysis must absolutely be done in two or more dimensions. Therefore, our analysis is two-dimensional by its very design. By pushing the capabilities of computer algebra systems to the limits, we have been able to extend this two-dimensional curl-preserving von Neumann stability analysis up to fourth order of accuracy.

Section 5 shows the results of this von Neumann stability analysis. We present such an analysis for globally curl-free WENO-like, P*N*P*M*-like and DG-like schemes to facilitate inter-comparison. In Sect. 5.1 the limiting CFL numbers for all these schemes are derived and documented in Tables 1, 2, and 3. We find, unsurprisingly, that WENO-like schemes offer the largest CFL numbers while DG-like schemes restrict us to substantially smaller CFL numbers. The P*N*P*M*-like schemes give us quite large CFL numbers at a much-reduced computational complexity. In Sect. 5.2 we document the dissipation and dispersion properties of the same three schemes. We do this for waves with wavelengths 5, 10, and 15 times the zone size. For each family of schemes, the dissipation and dispersion properties do indeed improve with increasing order of accuracy, as expected. This is shown in the figures associated with Sect. 5.2 and also in Tables 4, 5, and 6. We find that WENO-like schemes have dissipation and dispersion properties that are noticeably inferior to the DG-like schemes at the same order. However, we find that P*N*P*M*-like schemes have dissipation and dispersion properties that approach those of DG-like schemes at the same order while offering substantially larger CFL numbers.

Section 6 presents numerical results, where we show that our methods meet their design accuracies. We also show that with increasing order of accuracy the methods become very good at preserving quadratic energy. This is a welcome result, because the methods were not intentionally designed to preserve quadratic energy; yet they seem to do a good job. When the evolution of the PDE is curl-free, our methods also hold down the discrete circulation to machine accuracy over long integration times. The importance of this fact in the design of curl-preserving schemes is also discussed.

This paper has laid the essential foundation for several novel globally curl constraint-preserving methods and catalogued their many desirable properties. The next step would be to apply them to full PDE systems where their potential gains can be realized.

## Appendix A

Here we explicitly provide the nine coefficients of the 3 × 3 matrix “**A**” in Eq. (20). This should enable the reader to cross-check her or his mathematics. The first row is given by

$$ A_{1,1} = \frac{{\left( {\Delta x\,\cos \left( {\Delta y\,k_{y} } \right) - \Delta x} \right)\,\left| {{\text{v}}_{y} } \right| - {\text i}\,\Delta x\,\sin \left( {\Delta y\,k_{y} } \right)\,{\text{v}}_{y} + \left( {\Delta y\,\cos \left( {\Delta x\,k_{x} } \right) - \Delta y} \right)\,\left| {{\text{v}}_{x} } \right| - {\text i}\,\Delta y\,\sin \left( {\Delta x\,k_{x} } \right)\,{\text{v}}_{x} }}{\Delta x\,\Delta y}, $$

$$ A_{1,2} = - \frac{{{\text i}\,\sin \left( {\Delta x\,k_{x} } \right)\,\left| {{\text{v}}_{x} } \right| + \left( {1 - \cos \left( {\Delta x\,k_{x} } \right)} \right)\,{\text{v}}_{x} }}{2\,\Delta x}, $$

$$ A_{1,3} = - \frac{{\left( {{\text i}\,\sin \left( {\frac{{\Delta y\,k_{y} + \Delta x\,k_{x} }}{2}} \right) - {\text i}\,\sin \left( {\frac{{\Delta y\,k_{y} - \Delta x\,k_{x} }}{2}} \right)} \right)\,\left| {{\text{v}}_{y} } \right| + \left( {\cos \left( {\frac{{\Delta y\,k_{y} - \Delta x\,k_{x} }}{2}} \right) - \cos \left( {\frac{{\Delta y\,k_{y} + \Delta x\,k_{x} }}{2}} \right)} \right)\,{\text{v}}_{y} }}{2\,\Delta x}. $$

The second row is given by

$$ A_{2,1} = \frac{{6\,{\text i}\,\sin \left( {\Delta x\,k_{x} } \right)\,\left| {{\text{v}}_{x} } \right| + \left( {6 - 6\,\cos \left( {\Delta x\,k_{x} } \right)} \right)\,{\text{v}}_{x} }}{\Delta x}, $$

$$ A_{2,2} = \frac{{\left( {\cos \left( {\Delta y\,k_{y} } \right) - 1} \right)\,\left| {{\text{v}}_{y} } \right| - {\text i}\,\sin \left( {\Delta y\,k_{y} } \right)\,{\text{v}}_{y} + \left( { - 3\,\cos \left( {\Delta x\,k_{x} } \right) - 3} \right)\,\left| {{\text{v}}_{x} } \right| + 3\,{\text i}\,\sin \left( {\Delta x\,k_{x} } \right)\,{\text{v}}_{x} }}{\Delta x}, $$

$$ A_{2,3} = 0. $$

The third row is given by

$$ \begin{aligned} A_{3,1} & = \frac{{3\,\Delta x\,{\text{e}}^{{3\,{\text i}\,\Delta y\,k_{y} + \frac{{{\text i}\,\Delta x\,k_{x} }}{2}}} \,\left| {{\text{v}}_{y} } \right|}}{{\Delta y^{2} \,{\text{e}}^{{\frac{{3\,{\text i}\,\Delta y\,k_{y} }}{2} + {\text i}\,\Delta x\,k_{x} }} - \Delta y^{2} \,{\text{e}}^{{\frac{{3\,{\text i}\,\Delta y\,k_{y} }}{2}}} }} - \frac{{3\,\Delta x\,{\text{e}}^{{2\,{\text i}\,\Delta y\,k_{y} + \frac{{{\text i}\,\Delta x\,k_{x} }}{2}}} \,\left| {{\text{v}}_{y} } \right|}}{{\Delta y^{2} \,{\text{e}}^{{\frac{{3\,{\text i}\,\Delta y\,k_{y} }}{2} + {\text i}\,\Delta x\,k_{x} }} - \Delta y^{2} \,{\text{e}}^{{\frac{{3\,{\text i}\,\Delta y\,k_{y} }}{2}}} }} \\ & \quad - \frac{{3\,\Delta x\,{\text{e}}^{{{\text i}\,\Delta y\,k_{y} + \frac{{{\text i}\,\Delta x\,k_{x} }}{2}}} \,\left| {{\text{v}}_{y} } \right|}}{{\Delta y^{2} \,{\text{e}}^{{\frac{{3\,{\text i}\,\Delta y\,k_{y} }}{2} + {\text i}\,\Delta x\,k_{x} }} - \Delta y^{2} \,{\text{e}}^{{\frac{{3\,{\text i}\,\Delta y\,k_{y} }}{2}}} }} + \frac{{3\,\Delta x\,{\text{e}}^{{\frac{{{\text i}\,\Delta x\,k_{x} }}{2}}} \,\left| {{\text{v}}_{y} } \right|}}{{\Delta y^{2} \,{\text{e}}^{{\frac{{3\,{\text i}\,\Delta y\,k_{y} }}{2} + {\text i}\,\Delta x\,k_{x} }} - \Delta y^{2} \,{\text{e}}^{{\frac{{3\,{\text i}\,\Delta y\,k_{y} }}{2}}} }} \\ & \quad - \frac{{3\,\Delta x\,{\text{e}}^{{3\,{\text i}\,\Delta y\,k_{y} + \frac{{{\text i}\,\Delta x\,k_{x} }}{2}}} \,{\text{v}}_{y} }}{{\Delta y^{2} \,{\text{e}}^{{\frac{{3\,{\text i}\,\Delta y\,k_{y} }}{2} + {\text i}\,\Delta x\,k_{x} }} - \Delta y^{2} \,{\text{e}}^{{\frac{{3\,{\text i}\,\Delta y\,k_{y} }}{2}}} }} + \frac{{9\,\Delta x\,{\text{e}}^{{2\,{\text i}\,\Delta y\,k_{y} + \frac{{{\text i}\,\Delta x\,k_{x} }}{2}}} \,{\text{v}}_{y} }}{{\Delta y^{2} \,{\text{e}}^{{\frac{{3\,{\text i}\,\Delta y\,k_{y} }}{2} + {\text i}\,\Delta x\,k_{x} }} - \Delta y^{2} \,{\text{e}}^{{\frac{{3\,{\text i}\,\Delta y\,k_{y} }}{2}}} }} \\ & \quad - \frac{{9\,\Delta x\,{\text{e}}^{{{\text i}\,\Delta y\,k_{y} + \frac{{{\text i}\,\Delta x\,k_{x} }}{2}}} \,{\text{v}}_{y} }}{{\Delta y^{2} \,{\text{e}}^{{\frac{{3\,{\text i}\,\Delta y\,k_{y} }}{2} + {\text i}\,\Delta x\,k_{x} }} - \Delta y^{2} \,{\text{e}}^{{\frac{{3\,{\text i}\,\Delta y\,k_{y} }}{2}}} }} + \frac{{3\,\Delta x\,{\text{e}}^{{\frac{{{\text i}\,\Delta x\,k_{x} }}{2}}} \,{\text{v}}_{y} }}{{\Delta y^{2} \,{\text{e}}^{{\frac{{3\,{\text i}\,\Delta y\,k_{y} }}{2} + {\text i}\,\Delta x\,k_{x} }} - \Delta y^{2} \,{\text{e}}^{{\frac{{3\,{\text i}\,\Delta y\,k_{y} }}{2}}} }}, \\ \end{aligned} $$

$$ A_{3,2} = 0, $$

$$ A_{3,3} = - \frac{{\left( {3\,\cos \left( {\Delta y\,k_{y} } \right) + 3} \right)\,\left| {{\text{v}}_{y} } \right| - 3\,{\text i}\,\sin \left( {\Delta y\,k_{y} } \right)\,{\text{v}}_{y} + \left( {1 - \cos \left( {\Delta x\,k_{x} } \right)} \right)\,\left| {{\text{v}}_{x} } \right| + {\text i}\,\sin \left( {\Delta x\,k_{x} } \right)\,{\text{v}}_{x} }}{\Delta y}. $$

This completes our description of the nine coefficients of the 3 × 3 matrix “**A**” in Eq. (20).
